# A validated antibody toolbox for ALS research

**DOI:** 10.7554/eLife.111349

**Published:** 2026-11-03

**Authors:** Riham Ayoubi, Emma J MacDougall, Ian McDowell, Michael S Biddle, Bárbara T Ferreira, CongYao Zha, Marie-France Dorion, Jay P Ross, Sara Gonzalez Bolivar, Vera Ruiz Moleon, Charles Alende, Vincent Francis, Maryam Fotouhi, Mathilde Chaineau, Carol X Chen, Valerio EC Piscopo, Vincent Soubannier, Irina Shlaifer, Zhipeng You, Michael Nicouleau, Tracy Keates, Wen Hwa Lee, Brian D Marsden, Leonidas Koukouflis, Edvard Wigren, Carolyn A Marks, Luke M Healy, Patrick A Dion, Guy A Rouleau, Edward A Fon, Harvinder S Virk, Susanne Gräslund, Opher Gileadi, Aled M Edwards, Thomas M Durcan, Peter S McPherson, Carl Laflamme

**Affiliations:** 1 Structural Genomics Consortium, Montreal Neurological Institute-Hospital (The Neuro), McGill University Montreal Canada; 2 Neurodegenerative Diseases Research Group, Department of Neurology and Neurosurgery, Montreal Neurological Institute-Hospital (The Neuro), McGill University Montreal Canada; 3 NIHR Respiratory Biomedical Research Centre, Department of Respiratory Sciences, University of Leicester Leicester United Kingdom; 4 Early Drug Discovery Unit (EDDU), Department of Neurology and Neurosurgery, Montreal Neurological Institute-Hospital (The Neuro), McGill University Montreal Canada; 5 Department of Genetics, Stanford University School of Medicine Stanford United States; 6 Structural Genomics Consortium, Nuffield Department of Medicine, University of Oxford Oxford United Kingdom; 7 Centre for Human Genetics and Centre for Medicines Discovery, Nuffield Department of Medicine, University of Oxford Oxford United Kingdom; 8 Nuffield Department of Medicine, University of Oxford Oxford United Kingdom; 9 Structural Genomics Consortium, Department of Medicine, Karolinska Institute Solna Sweden; 10 Neuroimmunology Unit, Department of Neurology and Neurosurgery, Montreal Neurological Institute-Hospital (The Neuro), McGill University Montreal Canada; 11 School of Pharmaceutical Sciences, Faculty of Medicine, University of Ottawa Ottawa Canada; Universidade Federal do Rio de Janeiro Brazil; Goethe University Frankfurt Germany

**Keywords:** amyotrophic lateral sclerosis, ALS, antibody characterization, open science, protein expression profiling, Human

## Abstract

A substantial fraction of amyotrophic lateral sclerosis (ALS)-associated proteins remains poorly characterized, in part because of the limited availability of validated research antibodies. We established knockout (KO)-based antibody characterization workflows and demonstrated that widely used antibodies against the major ALS-associated protein C9orf72 lacked specificity (Laflamme et al., 2019). We subsequently scaled this framework to systematically benchmark research antibodies, revealing that up to 61% fail to perform as recommended by manufacturers (Ayoubi et al., 2023b). Here, we extend this approach by establishing the ALS-Reproducible Antibody Platform (ALS-RAP), a comprehensive effort to generate a publicly available dataset of KO-validated antibodies targeting proteins encoded by ALS risk genes. In total, we characterized 303 antibodies against 33 ALS-associated proteins to identify high-quality reagents for use in western blot, immunoprecipitation, and immunofluorescence. Using these antibodies, we profiled protein levels across human induced pluripotent stem cell-derived and primary neurological cell types. These analyses revealed diverse cellular distributions and higher levels of several ALS-associated proteins in glial populations, consistent with emerging evidence for immune contributions to ALS. Together, ALS-RAP provides a validated antibody toolbox and protein expression resource to support the study of ALS-associated proteins.

## Introduction

Research antibodies are among the most widely used tools for protein detection and quantification in biomedical research, yet their performance and specificity are often insufficiently characterized (Kahn et al., 2023; Biddle et al., 2024; Baker, 2015; Goodman, 2018). Building on our previous studies establishing knockout (KO)-based antibody characterization workflows and benchmarking antibody performance (Laflamme et al., 2019; Ayoubi et al., 2023b), we applied this framework to proteins encoded by amyotrophic lateral sclerosis (ALS) risk genes, a genetically defined but mechanistically heterogeneous disease. More than 30 genes have been linked to familial and sporadic ALS (Nijs and Van Damme, 2024; Rehm et al., 2015), yet most ALS-associated proteins remain poorly characterized at the protein level. This gap reflects both the limited availability of validated reagents and the lack of systematic protein-level characterization across relevant neurological cell types.

To address this limitation, we established the ALS-Reproducible Antibody Platform (ALS-RAP), a systematic effort to characterize antibodies targeting ALS-associated proteins using KO-based workflows (Ayoubi et al., 2025), with a focus on renewable reagents (monoclonal and recombinant antibodies). ALS-RAP builds on antibody characterization strategies developed through the Antibody Characterization through Open Science (YCharOS) initiative, an open-science ecosystem that brings together academic laboratories and antibody manufacturers to systematically evaluate research antibodies and openly disseminate characterization datasets (Monteiro et al., 2025; Lund-Johansen, 2023; Laflamme et al., 2021). To expand renewable reagent coverage, the Structural Genomics Consortium (SGC) generated recombinant antibodies for several ALS targets lacking suitable commercial reagents (Hornsby et al., 2015).

Through ALS-RAP, we assembled and characterized antibodies targeting 33 ALS-associated proteins and identified high-quality renewable reagents suitable for common applications. These validated antibodies enabled us to examine protein levels across human induced pluripotent stem cell (iPSC)-derived and primary neurological cell types. Together, this work extends antibody characterization to a disease-focused protein landscape and provides a resource for investigating these proteins in relevant cellular contexts.

## Results

### Genetic prioritization and initial characterization of ALS-RAP targets

We prioritized 33 ALS-related genes supported by replicated genetic evidence: *ACSL5*, *ALS2*, *ANG*, *ANXA11*, *ATXN2*, *C9orf72*, *CAV1*, *CCNF*, *CHCHD10*, *CHMP2B*, FIG4, *FUS*, *HNRNPA1*, *HNRNPA2B1*, *KIF5A*, *LGALS1*, *MATR3*, *NEK1*, *OPTN*, *PFN1*, *SETX*, *SIGMAR1*, *SOD1*, *SPG11*, *SQSTM1*, *TAF15*, *TARDBP*, *TBK1*, *TIA1*, *TUBA4A*, *UBQLN2*, *VAPB*, *VCP*. Building on a previous classification framework (Nijs and Van Damme, 2024), these genes were grouped according to variant frequency and estimated pathogenic impact, including rare variants with high, intermediate, or small effect sizes, as well as rare or common variants with uncertain impact. Of these, 25 genes have also been curated by the Clinical Genome Resource (ClinGen) ALS expert panel and classified as having evidence of disease association (Table 1; Rehm et al., 2015).

**Table 1.** ALS-associated genes selected for the ALS-RAP study.

Gene name	UniProt ID	Effect size (variants; effect size)	ClinGen curated description	PubMed entries (gene name +ALS)	PubMed entries (gene name)
*SOD1*	P00441	Rare; high	Definitive	4679	10,855
*TARDBP*	Q13148	Rare; high	Definitive	2846	5061
*C9orf72*	Q96LT7	Rare; high	Definitive	1952	2757
*FUS*	P35637	Rare; high	Definitive	1332	5694
*VCP*	P55072	Rare; high	Definitive	267	2354
*OPTN*	Q96CV9	Rare; high	Definitive	206	1640
*SQSTM1*	Q13501	Rare; intermediate	Moderate	183	3848
*TBK1*	Q9UHD2	Rare; high	Definitive	178	2749
*VAPB*	O95292	Rare; intermediate	Definitive	169	417
*ATXN2*	Q99700	Rare; intermediate	Definitive	163	437
*UBQLN2*	Q9UHD9	Rare; high	Definitive	139	206
*ALS2*	Q96Q42	Rare; intermediate	Definitive	127	250
*ANG*	P03950	Rare; intermediate	Limited	99	1592
*SETX*	Q7Z333	Rare; intermediate	Not available	93	257
*CHCHD10*	Q8WYQ3	Rare; intermediate	Moderate	93	174
*HNRNPA1*	P09651	Rare; unknown	Not available	91	574
*PFN1*	P07737	Rare; intermediate	Definitive	79	356
*TAF15*	Q92804	Rare; intermediate	Limited	66	251
*NEK1*	Q96PY6	Rare; high	Definitive	64	192
*MATR3*	P43243	Rare; unknown	Definitive	61	162
*KIF5A*	Q12840	Rare; intermediate	Definitive	61	259
*CHMP2B*	Q9UQN3	Rare; intermediate	Definitive	48	225
*TIA1*	P31483	Rare; intermediate	Limited	47	1146
*ANXA11*	P50995	Rare; intermediate	Definitive	47	124
*CCNF*	P41002	Rare; small	Limited	41	184
*FIG4*	Q92562	Rare; intermediate	Limited	40	170
*HNRNPA2B1*	P22626	Rare; unknown	Moderate	38	535
*SPG11*	Q96JI7	Rare; intermediate	Definitive	34	283
*TUBA4A*	P68366	Rare; high	Moderate	32	95
*SIGMAR1*	Q99720	Rare; unknown	Not available	27	202
*CAV1*	Q03135	Rare; small	Limited	14	6065
*ACSL5*	Q9ULC5	Common; small	Not available	4	192
*LGALS1*	P09382	Rare; high	Not available	3	308

To estimate how ALS-associated proteins are represented in the literature, PubMed searches were performed for each gene using the gene name alone or combined with “ALS” (Table 1, ordered by number of PubMed entries). As shown in a line-scan plot (Figure 1—figure supplement 1), four genes—*SOD1*, *TARDBP*, *C9orf72*, and *FUS*—dominate the ALS literature, accounting for 10,809 of 13,173 publications (>80%). In contrast, the remaining 29 genes are far less studied in ALS and collectively define the “dark ALS-ome,” referring to ALS-associated proteins with limited functional characterization. Notably, seven of these genes—*VCP*, *OPTN*, *SQSTM1*, *TBK1*, *ANG*, *TIA1*, and *CAV1*—have been investigated in other biological contexts.

### Reagent evaluation and antibody development for ALS-RAP targets

According to the CiteAb database (citeab.com), only 3 of the 33 ALS-RAP targets have a top-cited renewable antibody characterized using genetic knockdown or KO approaches, highlighting a substantial gap in validated reagents. We therefore assessed renewable antibody availability across all targets prior to benchmarking with YCharOS characterization workflows, as batch-to-batch variability of polyclonal antibodies limits reproducibility (Ayoubi et al., 2023b; Marx, 2020). Six proteins—ANXA11, OPTN, MATR3, PFN1, UBQLN2, and VCP—had limited commercially renewable antibodies. To address this gap, the SGC generated recombinant single-chain variable fragment (scFv) antibodies optimized to recognize native, folded proteins, making them particularly well suited for IP (Preger et al., 2020). Recombinant full-length proteins or protein domains were produced, purified, site-specifically biotinylated, and used as antigens for phage-display selections. Individual scFv clones were initially screened by ELISA, homogeneous time-resolved fluorescence, and suspension bead assays to confirm target binding and specificity. Candidate binders were subsequently evaluated by immunoprecipitation coupled to mass spectrometry (IP-MS) using established protocols (Marcon et al., 2015), with at least three scFv clones screened for each target. The top-performing clone, selected based on IP-MS target enrichment, was then benchmarked alongside commercially available antibodies in the YCharOS validation pipeline. Antibody-encoding plasmids have been deposited at Addgene to ensure community access, and the amino acid sequences of reported scFvs are provided in Supplementary file 2.

In parallel, we evaluated KO cell line availability for antibody characterization. Twenty-one commercial KO lines were available from YCharOS partners and were selected based on parental expression levels above 2.5 log₂(TPM+1) in the DepMap database, a threshold suitable for antibody characterization studies (Ayoubi et al., 2025). To complete coverage, 12 additional custom KO lines were generated and are available upon request, while *VCP* was targeted with siRNA as it is an essential gene.

### Antibody performance and coverage for ALS-RAP proteins

**Figure 1.**
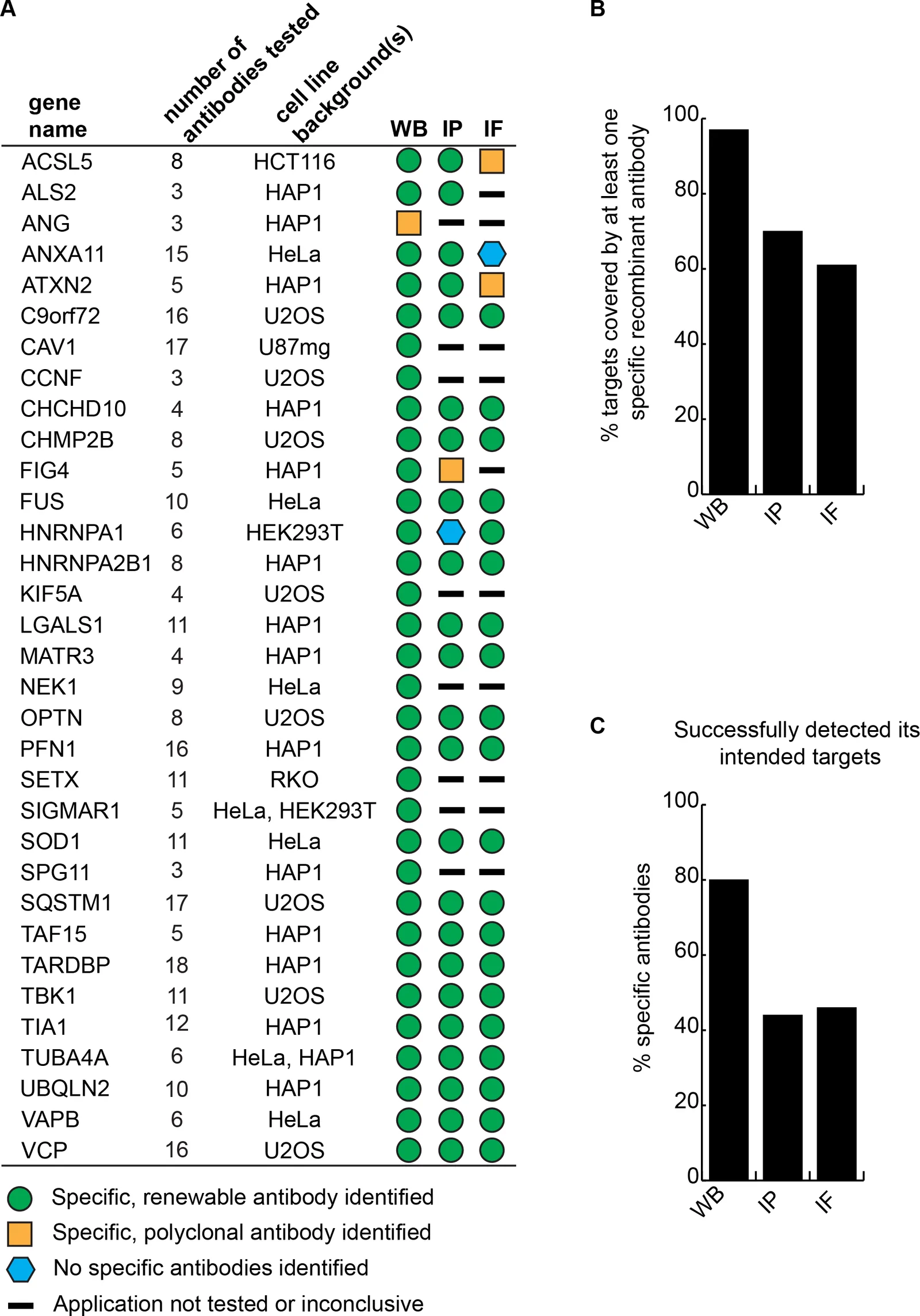
Summary of antibody performance for ALS-RAP proteins. (**A**) Overview of the identification of high-quality antibodies targeting ALS-RAP proteins. (**B**) Percentage of ALS-RAP targets covered by at least one specific renewable (monoclonal or recombinant) antibody for the indicated application. (**C**) Percentage of tested antibodies that met specificity criteria in each indicated application.

Antibody performance for ALS-RAP targets was systematically evaluated in western blot (WB), IP, and immunofluorescence (IF) using KO-based characterization protocols (Ayoubi et al., 2025). In total, 303 antibodies (297 commercial and 6 custom scFv antibodies) targeting 33 ALS-associated proteins were tested. All antibodies were assessed in WB. For five proteins—ALS2, FIG4, NEK1, SIGMAR1, and SPG11—the absence of a specific antibody precluded confident assessment of antibody performance, as we could not determine whether this reflected uniformly poor antibody performance or suboptimal IF conditions for these targets. These datasets were therefore not included in Zenodo reports. Antibodies against ANG, CAV1, CCNF, KIF5A, and SETX were not evaluated in IP and/or IF due to the recent availability of renewable reagents, specialized experimental requirements (e.g., CCNF and cell-cycle arrest), or the secreted nature of ANG (Figure 1A).

**Figure 1—figure supplement 1.**
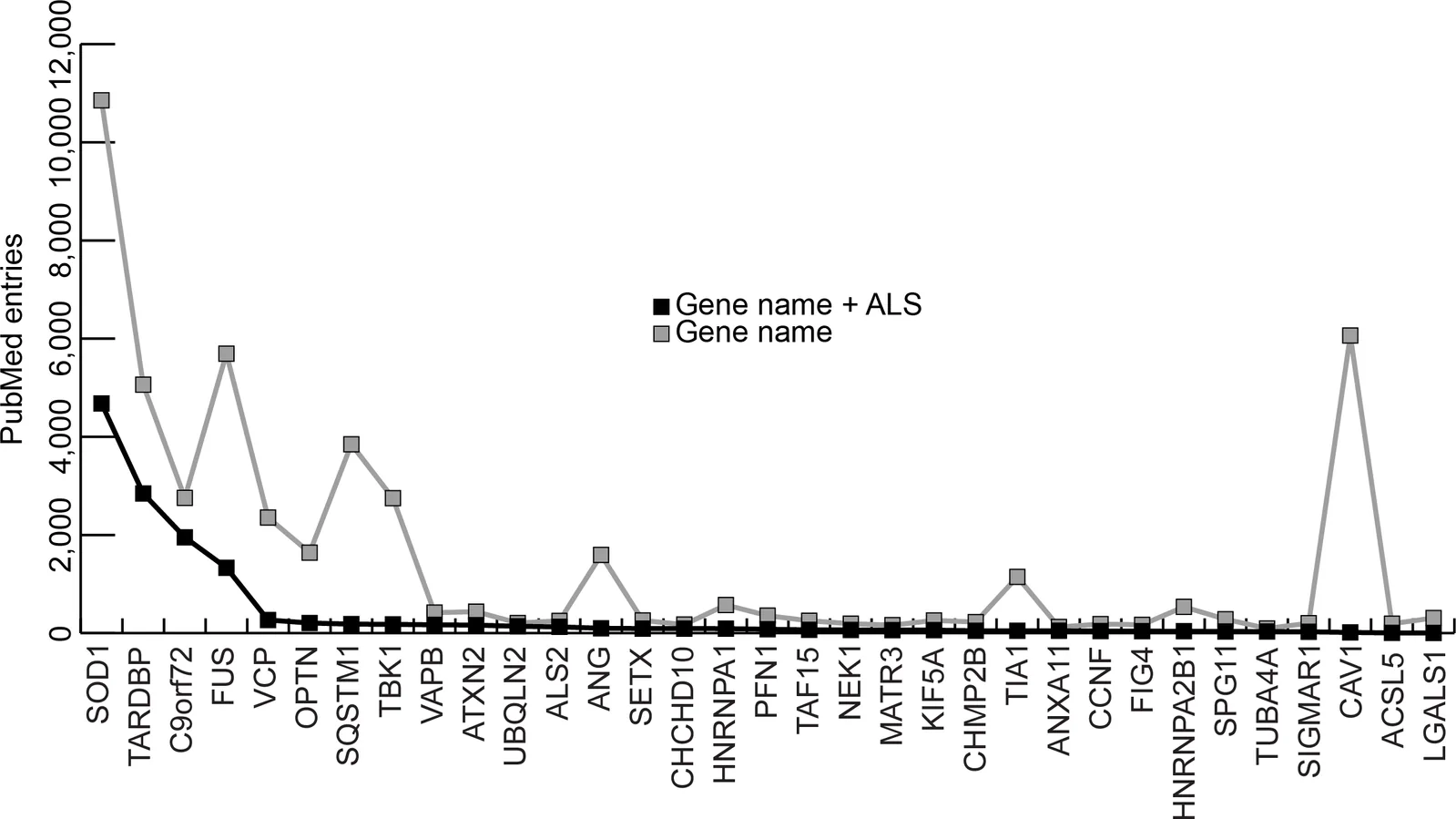
Characterization of ALS-RAP targets. Number of PubMed entries related to ALS-RAP genes. Black points represent the number of entries retrieved using the search term “gene name + ALS,” while gray points represent entries retrieved using the gene name alone. The x-axis indicates the number of PubMed entries, and the y-axis lists the gene names.

**Figure 1—figure supplement 2.**
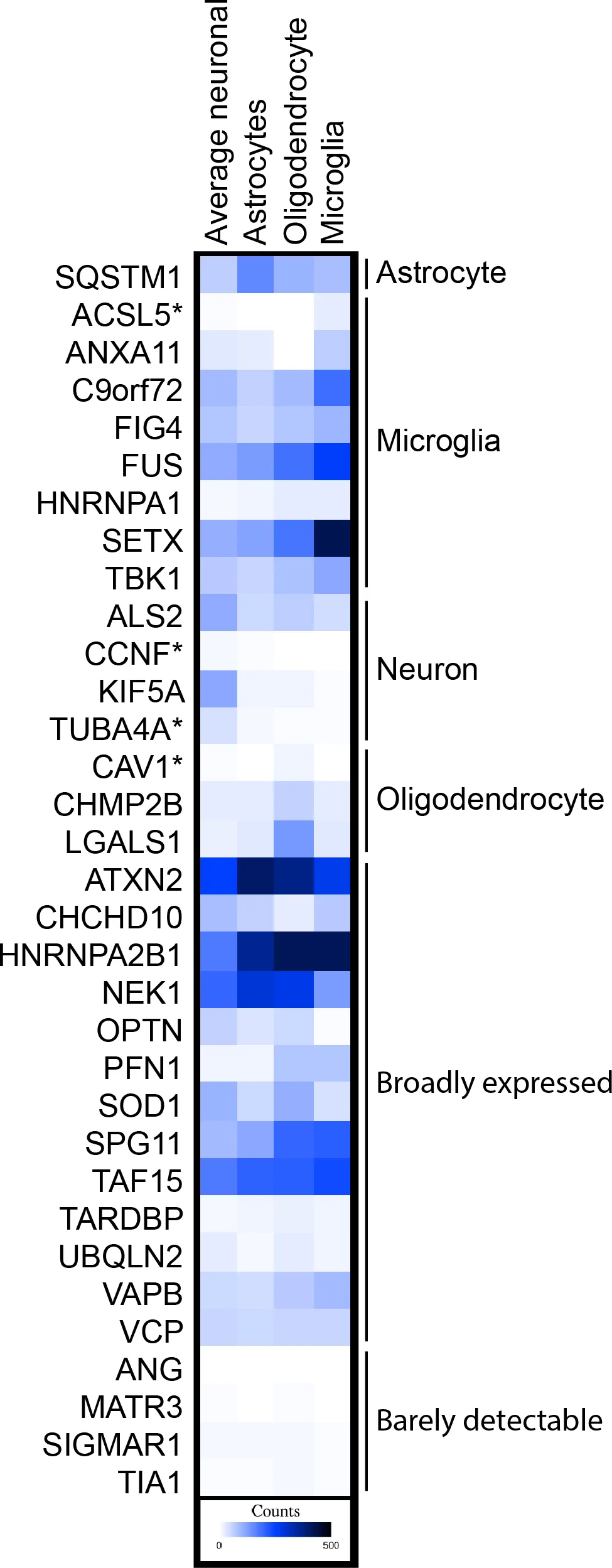
ALS-RAP RNA expression across brain cell types. Heatmap showing normalized RNA expression values from the Human Protein Atlas (HPA) single-nuclei brain dataset. Expression is given as nTPM values on a scale from 0 to 500. Genes denoted with an asterisk (*) are classified as “barely detectable” but show substantial expression in a single-cell type.

**Figure 1—figure supplement 3.**
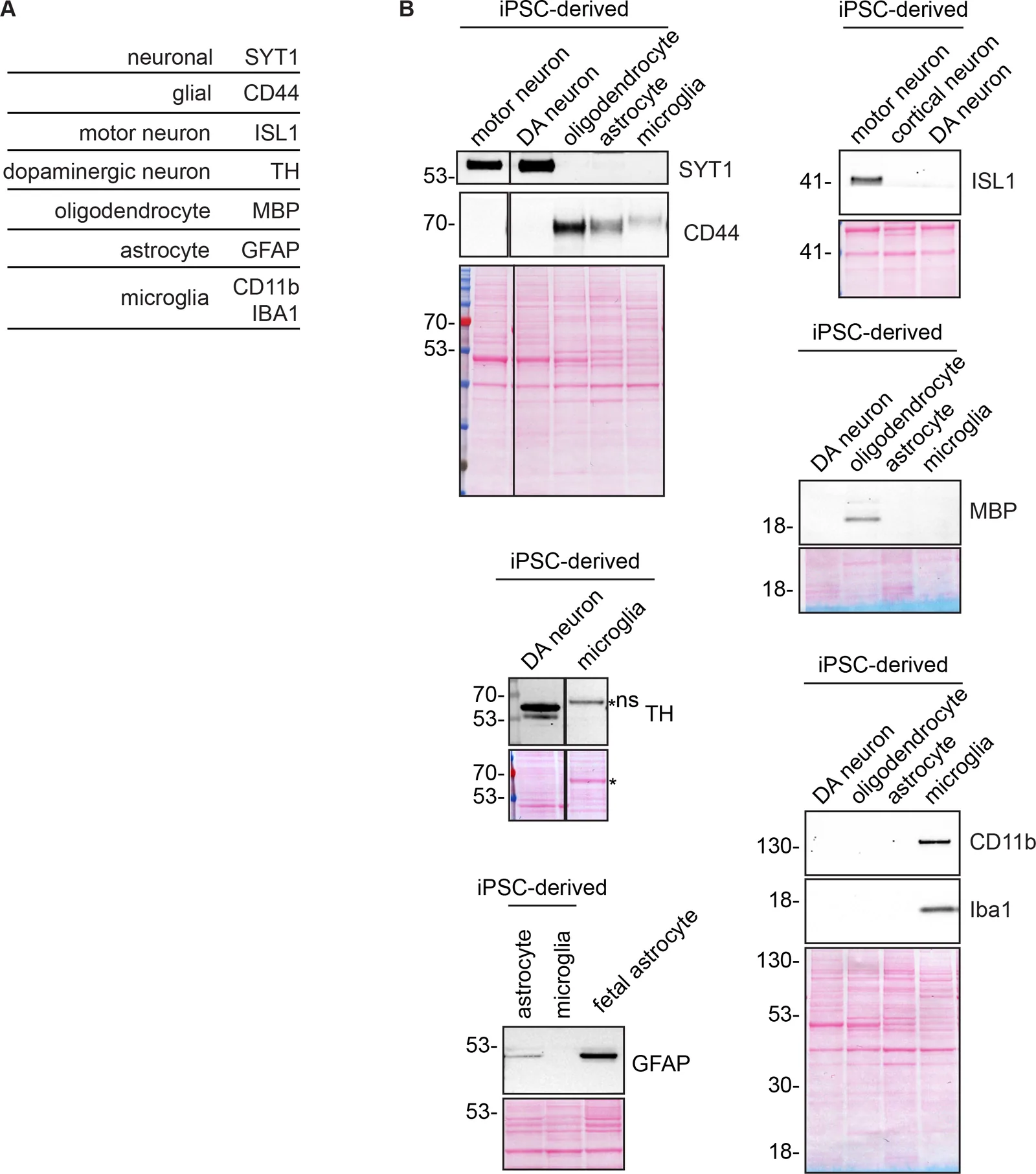
Protein-level characterization of iPSC-derived neurological cells. (**A**) Table listing cell type–specific marker proteins. (**B**) WB analysis of marker proteins using antibodies against the indicated proteins. A Ponceau-stained membrane is shown to assess protein loading and transfer efficiency. Lysates were prepared from the indicated human iPSC-derived cell types or fetal astrocyte culture. Vertical lines between the motor neuron and DA neuron lanes and between the DA neuron and microglia lanes indicate that WB were spliced to remove additional conditions not relevant to this study. DA, dopaminergic; NPC, neuronal progenitor cells.

**Figure 1—figure supplement 4.**
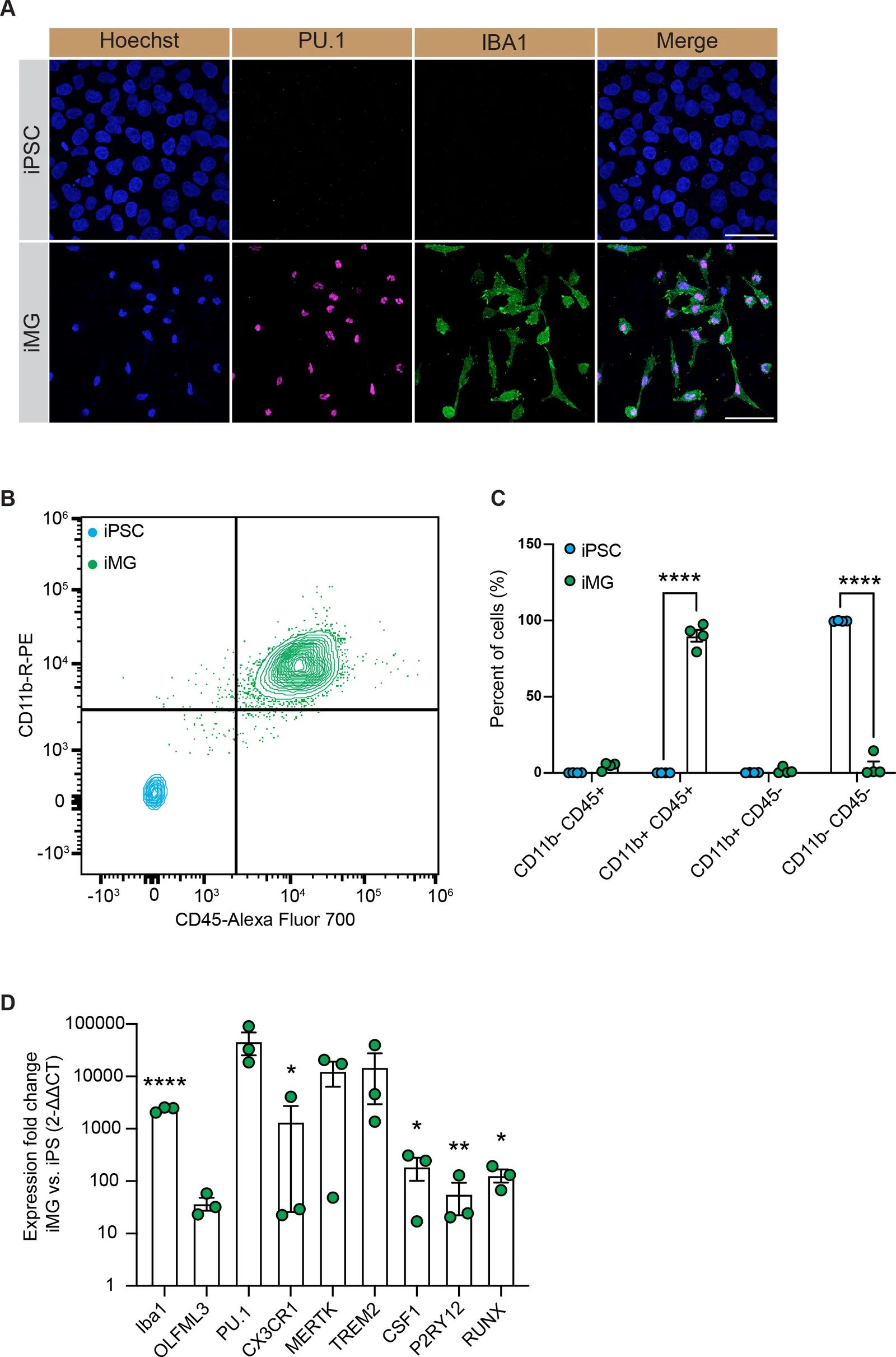
Quality control and marker validation of iPSC-derived microglia. (**A**) Immunofluorescence staining showing expression of IBA1 and PU.1 in iMGs, with no detectable signal in undifferentiated iPSCs. (**B**) Representative flow cytometry plots demonstrating expression of CD45 and CD11b in iMGs but not in iPSCs. (**C**) Quantification of the percentage of CD45-positive, CD11b-positive, and double-positive cells in iPSCs and iMGs, as measured by flow cytometry (N=4). Statistical analysis was performed using two-way ANOVA with Bonferroni post hoc test. (**D**) RT-qPCR expression levels of canonical microglial markers in iMGs compared to iPSCs (N=3). Statistical analysis was performed using Student’s *t*-test. iMG, iPSC-derived microglia; iPSC, induced pluripotent stem cell. *p≤0.05, **p≤0.01, ***p≤0.001, ****p≤0.0001. Scale bar, 50 µm.

**Figure 1—figure supplement 5.**
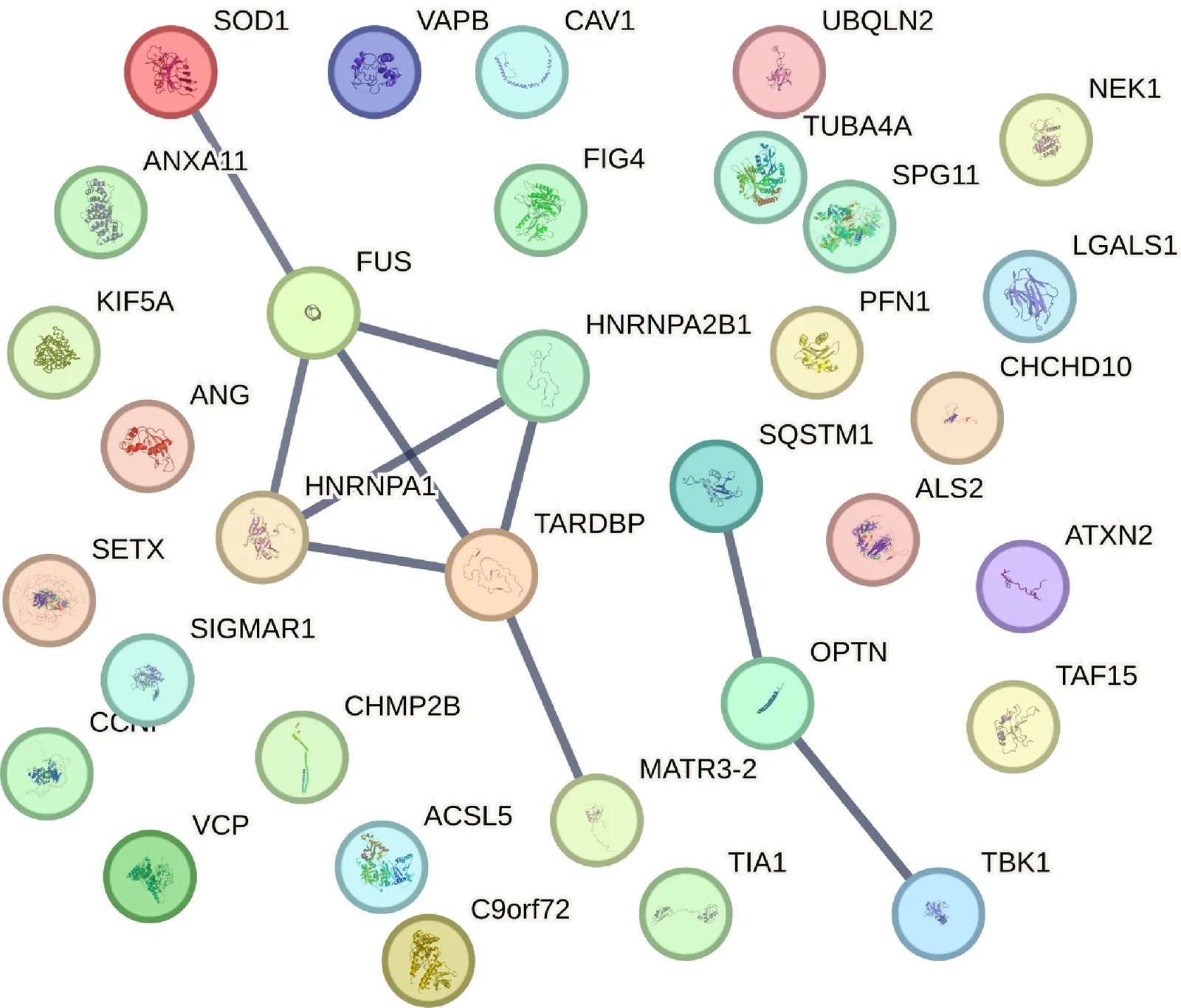
STRING analysis of known physical interactions between ALS-RAP proteins.

Antibodies were evaluated independently for each application as previously described (Ayoubi et al., 2023b). In WB, antibodies were considered specific when an immunoreactive band was absent in the corresponding KO lysate. In IP, antibodies were considered successful when the target protein was specifically enriched relative to the starting material, as confirmed by immunoblot detection of the immunoprecipitated protein. In IF, antibodies were considered specific when the mean fluorescence intensity in wild-type cells was at least 1.5-fold higher than that measured in KO cells using the mosaic WT/KO assay. For IF, a minimum of 250 WT and 250 KO cells were quantified for each antibody. Detailed experimental workflows have been described previously (Ayoubi et al., 2025).

Across all 33 targets, at least one renewable, KO-validated antibody was identified for 32 (97%), 23 (70%), and 20 (61%) proteins in WB, IP, and IF, respectively (Figure 1B). Overall, 243 of 303 antibodies (80%) detected their intended target in WB, 105 of 238 (44%) immunoprecipitated their target, and 107 of 231 (46%) demonstrated specific target staining in IF (Figure 1C).

The newly developed scFv antibodies filled key gaps; for example, the scFv against PFN1 was the only antibody—among 16 tested—capable of immunoprecipitating the protein (Ayoubi et al., 2023a). Collectively, these results demonstrate that renewable, KO-validated antibodies cover most ALS-associated proteins, enabling reproducible studies of ALS biology.

For transparency and reuse, we generated 33 antibody characterization reports (one per protein), reviewed with YCharOS industry partners and deposited on Zenodo, with a subset published through the F1000Research YCharOS Gateway (Biddle and Virk, 2023). All antibodies tested are listed in Supplementary file 1 with links to their corresponding characterization reports. Research Resource Identifiers (RRIDs) were obtained for all antibodies and KO cell lines to facilitate reagent traceability (Bandrowski et al., 2023; Bairoch, 2018). Antibodies used for downstream protein detection analyses were selected based on these characterization data, prioritizing renewable reagents. Catalogue numbers for these antibodies are provided in the ‘Key resources table’.

### Antibody characterization case study: Galectin-1

To illustrate the YCharOS antibody characterization workflow, we present the characterization of antibodies targeting Galectin-1, encoded by the *LGALS1* gene. Galectin-1 is a lectin implicated in immune and stress responses in the nervous system and has been reported in association with ALS in transcriptomic and functional studies (Cai et al., 2022; Kato et al., 2001). Antibodies were not assigned scores, consistent with the YCharOS philosophy that end users should evaluate raw data to select fit-for-purpose reagents based on their specific experimental needs.

Using DepMap transcriptomic data, the HAP1 cell line was selected for antibody characterization because it expresses *LGALS1* at adequate levels (7.9 log₂(TPM+1)), above the 2.5 threshold used for antibody characterization studies (Ayoubi et al., 2023b). An *LGALS1* KO HAP1 cell line was obtained from Revvity, and 10 renewable commercial antibodies from YCharOS partners were assembled for benchmarking (Table 2).

**Table 2.** Galectin-1 antibodies tested in this study.

Company	Catalog number	Lot number	RRID (antibody registry)	Clonality	Clone ID	Host	Concentration (µg/µL)	Vendors recommended applications
Abcam	ab108389*	1018000-29	AB_10863417	Recombinant mono	EPR3205	Rabbit	1.35	WB, IF
Abcam	ab138513^†^	1086382-8	AB_2894851	Monoclonal	EPR3206(2)	Rabbit	0.26	WB, IF
Abcam	ab192842*	1011456-2	AB_3725515	Recombinant mono	SP247	Rabbit	0.04	WB
ABclonal	A21218*	3522042922	AB_3725523	Recombinant mono	ARC53156	Rabbit	1.20	WB
Aviva Systems Biology	ARP58491_P050	QC25810-42978	AB_2046045	Polyclonal	-	Rabbit	0.50	WB
Bio-Techne (R&D Systems)	MAB1152^†^	CJKC0325011	AB_3657585	Monoclonal	933133	Mouse	0.50	WB
Cell Signaling Technology	12936*	3	AB_2798065	Recombinant mono	D608T	Rabbit	0.10	WB, IF
Thermo Fisher Scientific	MA5-32779*	79527780	AB_2810056	Recombinant mono	JM13-37	Rabbit	1.00	WB, IP, IF
Thermo Fisher Scientific	MA5-35566*	79531623	AB_2849466	Recombinant mono	7W5Q6	Rabbit	2.00	WB, IF
Thermo Fisher Scientific	MA5-47904*	79531680	AB_3074348	Recombinant mono	SAIC-25B-112	Rabbit	1.00	WB

* Recombinant antibody.

† Monoclonal antibody.

**Figure 2.**
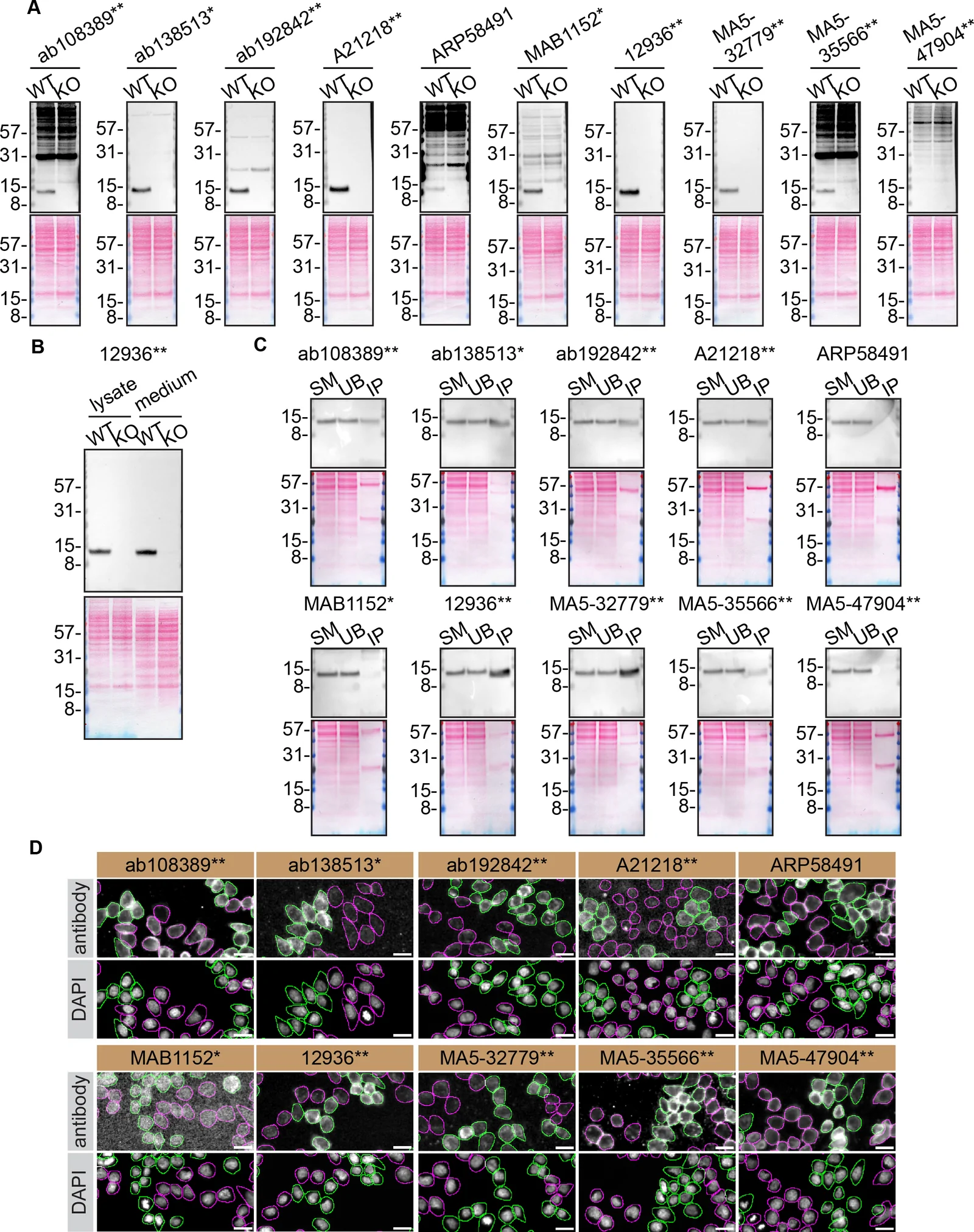
YCharOS antibody characterization workflow for Galectin-1 (*LGALS1*). Ten Galectin-1 antibodies were evaluated side-by-side across three applications using HAP1 WT and *LGALS1* KO cells. (**A**) Whole-cell protein lysates from HAP1 WT and *LGALS1* KO cells (20 µg per lane) were analyzed in WB using the indicated Galectin-1 antibodies. Ponceau-stained membranes are shown to assess protein loading and transfer efficiency. (**B**) Whole-cell protein lysates (10 µg) and conditioned media (10 µg) from HAP1 WT and *LGALS1* KO cells were analyzed in WB using antibody 12936, as in (**A**), to detect intracellular and secreted Galectin-1. (**C**) Immunoprecipitation was performed from HAP1 WT cell lysates using the indicated Galectin-1 antibodies. Starting material (SM), unbound fractions (UB), and whole immunoprecipitates (IP) were analyzed by WB. Anti-Galectin-1 antibody A21218 was used as a primary antibody. (**D**) HAP1 WT and *LGALS1* KO cells were differentially labeled with green and far-red fluorescent dyes, respectively, mixed at a 1:1 ratio, and plated in optically clear 96-well plates. Cells were stained with the indicated Galectin-1 antibodies, followed by Coralite 555–conjugated secondary antibodies and DAPI. Images were acquired in the blue (DAPI), green (WT), red (antibody staining), and far-red (KO) channels. Representative grayscale images of the blue and red channels are shown; WT and KO cells are outlined with green and magenta dashed lines, respectively. Scale bar, 10 µm. Recombinant antibodies are denoted by double asterisks (**) and monoclonal antibodies by single asterisks (*) after the catalogue numbers; polyclonal antibodies are unmarked. Antibody screening was performed in two independent experiments; representative data from one experiment are shown. Galectin-1 antibody characterization raw datasets are available at https://doi.org/10.5281/zenodo.21538630.

Antibody performance was evaluated across WB, IP, and IF using WT and KO HAP1 cells. In WB experiments, high-quality antibodies detected Galectin-1 in WT lysates with complete signal loss in KO lysates (Figure 2A). Galectin-1 was also detected in conditioned medium, consistent with its known secretion (Figure 2B). In IP experiments, antibodies were assessed for their ability to enrich Galectin-1 from cell lysates, with enrichment in the IP fraction and partial depletion from the unbound fraction indicating effective capture (Figure 2C). Finally, antibody performance in IF was evaluated using a mosaic strategy in which fluorescently labeled WT and KO cells were mixed and imaged in the same field of view, minimizing staining and imaging bias (Figure 2D). Complete experimental datasets and antibody characterization reports are available through Zenodo.

### ALS-RAP antibody recommendations on the OGA website

**Figure 3.**
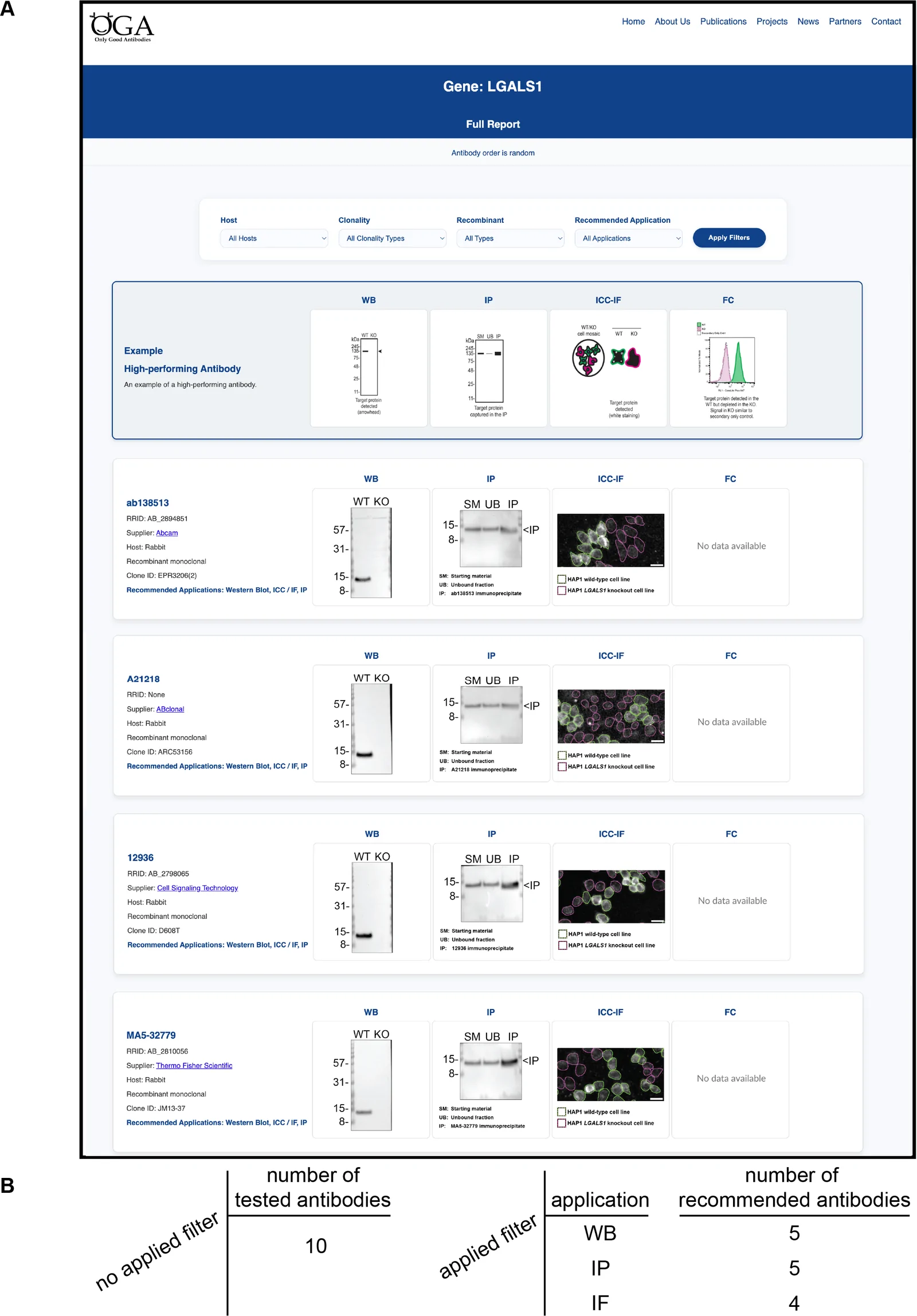
Accessing curated antibody characterization data through the OGA website. YCharOS antibody characterization data for ALS-RAP proteins are accessible through the Only Good Antibodies (OGA) website (https://onlygoodantibodies.co.uk/). The OGA homepage provides a searchable interface for the 33 ALS-RAP proteins, together with 35 additional neurological disease-associated proteins evaluated by YCharOS, and includes links to key OGA publications, data interpretation guidelines, antibody characterization protocols, and a portal for nominating new targets. (**A**) Representative results page generated by searching *LGALS1*, showing filters for host species, clonality, recombinant format, and OGA-recommended applications; the WB filter is applied. A schematic panel at the top illustrates ideal antibody performance across applications, while antibodies matching the selected filters are displayed in randomized order. (**B**) Number of antibodies returned following application of the selected filters.

In addition to dissemination through YCharOS, ALS-RAP antibody characterization datasets were independently evaluated by the Only Good Antibodies (OGA) initiative (Biddle et al., 2024), which did not participate in generating the experimental data. OGA integrates KO-based characterization datasets from YCharOS into a publicly accessible antibody assessment platform (https://onlygoodantibodies.co.uk/), where reagents are curated and presented to facilitate antibody selection by end users. Antibody entries include direct links to manufacturer purchasing pages. A screenshot of the Galectin-1 dataset illustrates the interface: a schematic representing high-quality antibodies is shown at the top, while all tested antibodies are listed below with application-specific recommendations derived from OGA analysis of the YCharOS data (Figure 3).

By adding an interpretive layer to the raw characterization datasets, OGA improves the discoverability of validated antibodies against ALS-associated proteins while preserving full access to the underlying data. This integration complements the ALS-RAP resource by pairing systematic KO-based characterization with a user-oriented recommendation framework.

### Neurological expression landscape of ALS-RAP proteins

#### Cellular context for ALS-RAP expression analysis

**Figure 4.**
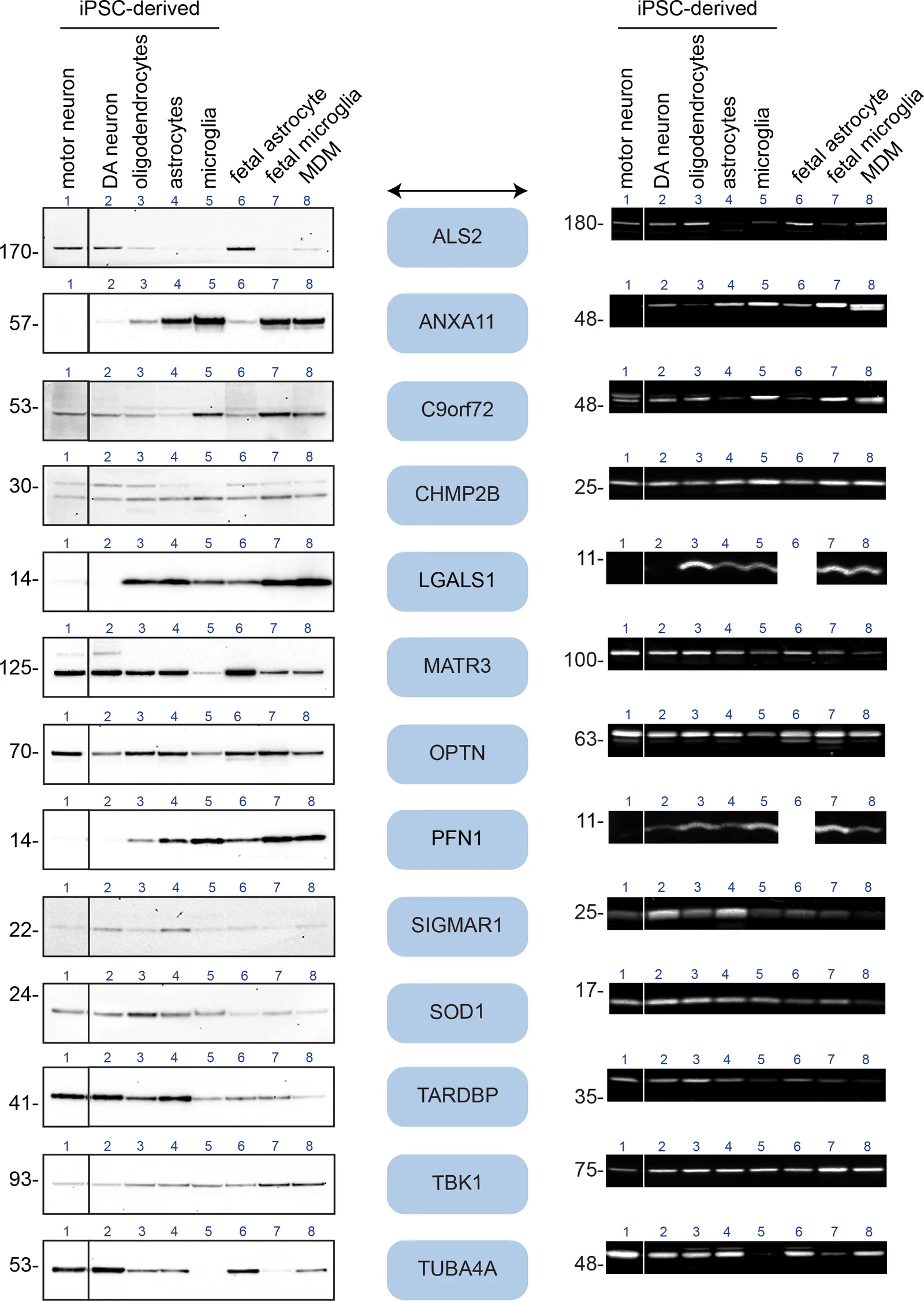
Detection of 13 ALS-RAP proteins across five human-derived neurological cell types and three primary cell types. WB showing detection of 13 ALS-RAP proteins in eight lysates from the indicated five iPSC-derived cell types and three primary cell types. For each protein, two independent experiments are shown: chemiluminescence-based WB (left), performed using a defined lysate batch across all corresponding blots, and fluorescence-based WB (right), performed using a second independent lysate batch. Proteins are listed in alphabetical order. Vertical lines between the motor neuron and DA neuron lanes indicate that the blots were spliced to remove additional conditions not relevant to this study. Lane identities: 1, motor neurons; 2, DA neurons; 3, oligodendrocytes; 4, astrocytes; 5, microglia; 6, fetal astrocytes; 7, fetal microglia; 8, MDMs. DA, dopaminergic; MDM, monocyte-derived macrophages. Raw WB data and supporting working sheet for the ALS-RAP protein expression profiling across neurological cell types by chemiluminescent WB are available at https://doi.org/10.5281/zenodo.18484385. Raw WB data and supporting working sheet for the ALS-RAP protein expression profiling across neurological cell types by fluorescent WB are available at https://doi.org/10.5281/zenodo.18484483.

To provide context for protein expression analyses, we first examined RNA expression of ALS-associated genes using the Human Protein Atlas (HPA) single-nucleus brain dataset (Karlsson et al., 2021). Genes were categorized as neuron-enriched, glial-enriched (astrocytes, oligodendrocytes, and microglia), or broadly expressed based on relative RNA enrichment across cell types. Several genes showed preferential neuronal expression (*ALS2*, *CCNF*, *KIF5A*, and *TUBA4A*), whereas others were enriched in glial populations (*ACSL5*, *ANXA11*, *C9orf72*, *CAV1*, *CHMP2B*, FIG4, *FUS*, *HNRNPA1*, *LGALS1*, *SETX*, *SQSTM1*, and *TBK1*). The remaining genes were broadly expressed, while four (*ANG*, *MATR3*, *SIGMAR1*, and *TIA1*) were barely detected across neurological cell types (Figure 1—figure supplement 2). Together with previously published single-cell RNA sequencing datasets of the human brain (McKeever et al., 2025), these analyses highlight the heterogeneous expression of ALS-associated genes.

Because RNA abundance does not necessarily predict protein levels (Gygi et al., 1999; Vogel and Marcotte, 2012), we next examined ALS-RAP protein expression across neurological cell types. Human iPSC-derived motor neurons, dopaminergic neurons, oligodendrocytes, astrocytes, and microglia were analyzed, complemented with primary human fetal astrocytes, fetal microglia, and monocyte-derived macrophages (MDMs). Cell identity was confirmed using established marker analyses (Figure 1—figure supplements 3 and 4).

#### Protein profiling across neurological cell types

**Figure 5.**
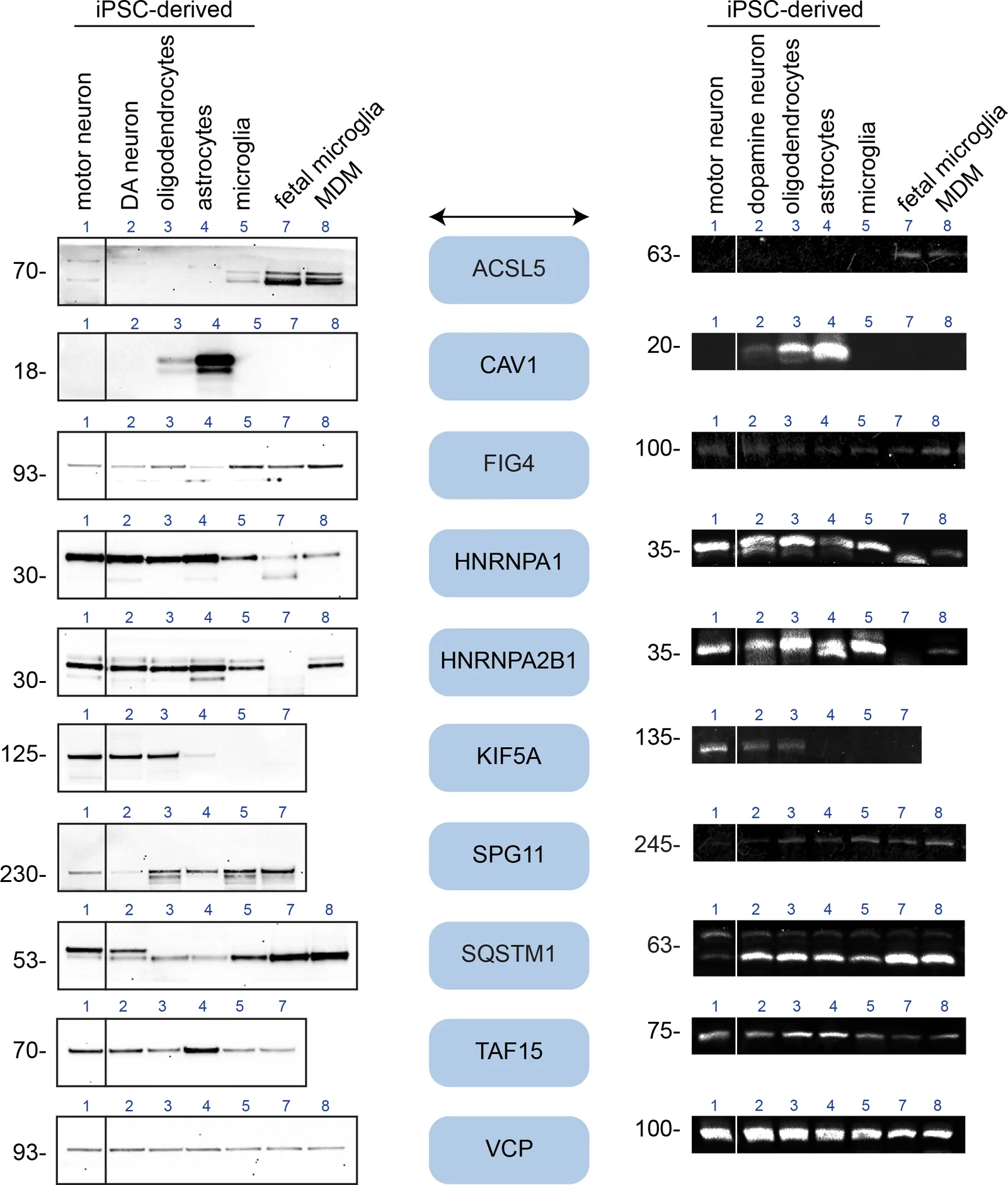
Detection of 10 ALS-RAP proteins across five human-derived neurological cell types and two primary cell types. WB showing detection of 10 ALS-RAP proteins in 7 lysates from the indicated five iPSC-derived cell types and 2 primary cell types. Fetal astrocyte lysate was not available for either chemiluminescence-based WB (left) and fluorescence-based WB (right). Proteins are listed in alphabetical order. Lane identities: 1, motor neurons; 2, DA neurons; 3, oligodendrocytes; 4, astrocytes; 5, microglia; 7, fetal microglia; 8, MDMs. DA, dopaminergic; MDM, monocyte-derived macrophages. Raw WB data and supporting working sheet for the ALS-RAP protein expression profiling across neurological cell types by chemiluminescent WB are available at https://doi.org/10.5281/zenodo.18484385. Raw WB data and supporting working sheet for the ALS-RAP protein expression profiling across neurological cell types by fluorescent WB are available at https://doi.org/10.5281/zenodo.18484483.

**Figure 6.**
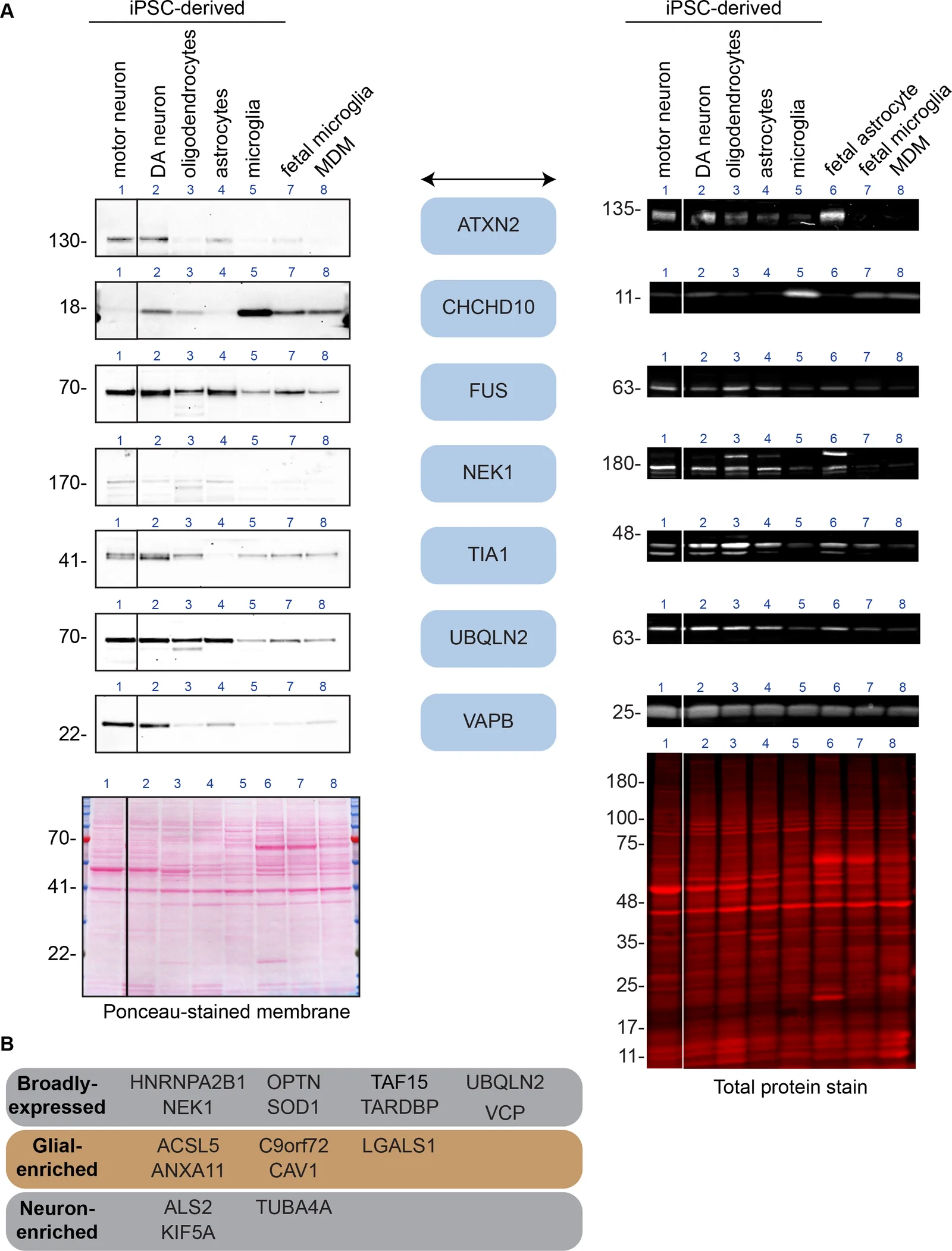
Detection of seven ALS-RAP proteins across five human-derived neurological cell types and primary cell types. (**A**) Chemiluminescence-based WB (left) showing detection of seven ALS-RAP proteins in seven lysates from the indicated five iPSC-derived cell types and two primary cell types. Fetal astrocyte lysate was not available for this WB. Fluorescence-based WB (right) showing detection of seven ALS-RAP proteins in eight lysates from the indicated five iPSC-derived cell types and three primary cell types. A representative Ponceau-stained membrane and total protein stain of all eight samples are shown to exemplify protein loading and transfer efficiency. Lane identities: 1, motor neurons; 2, DA neurons; 3, oligodendrocytes; 4, astrocytes; 5, microglia; 6, fetal astrocytes; 7, fetal microglia; 8, MDMs. DA, dopaminergic; MDM, monocyte-derived macrophages. (**B**) Summary of 16 targets for which WB data and Human Protein Atlas RNA expression profiles are concordant, grouped by expression category. Raw WB data and supporting working sheet for the ALS-RAP protein expression profiling across neurological cell types by chemiluminescent WB are available at https://doi.org/10.5281/zenodo.18484385. Raw WB data and supporting working sheet for the ALS-RAP protein expression profiling across neurological cell types by fluorescent WB are available at https://doi.org/10.5281/zenodo.18484483.

We profiled 30 ALS-RAP proteins in WB using two independent preparations of the same cell types: one analyzed by chemiluminescence-based detection and the other by fluorescence-based WB (Figures 4—6). For each target, the best-performing renewable antibody identified during YCharOS screening was used. KO-validated primary bands were consistently detected at the expected molecular weight across neurological cell types. Additional bands were occasionally observed (e.g., MATR3, SQSTM1, ANXA11, ACSL5, UBQLN2) but were not interpreted, as we could not determine whether they corresponded to the intended target or reflected antibody cross-reactivity. Three proteins were excluded from expression profiling: CCNF (requires cell-cycle arrest for detection), ANG (secreted protein not readily detected in lysates), and SETX (insufficient signal-to-noise). Relative signal intensities within each detection modality were compared to identify general patterns of protein detection across neuronal and glial populations.

Many ALS-RAP proteins were detected across both neuronal and glial cell types, including ALS2, CHMP2B, FIG4, FUS, HNRNPA1, HNRNPA2B1, MATR3, NEK1, OPTN, SIGMAR1, SOD1, SQSTM1, TAF15, TARDBP, TBK1, TIA1, UBQLN2, and VCP. Several proteins showed higher signal in glial populations in both datasets, including ACSL5, ANXA11, C9orf72, CAV1, CHCHD10, LGALS1, PFN1, and SPG11. ACSL5, ANXA11, C9orf72, and CHCHD10 were detected at higher levels in microglial populations, while CAV1 showed stronger signal in astrocytes. These patterns were consistent between iPSC-derived and primary glial cells. In contrast, several proteins showed higher protein levels in neuronal populations. ATXN2 was strongly detected in motor and dopaminergic neurons, while KIF5A and TUBA4A were enriched in neuronal lineages. VAPB was primarily detected in neurons, with additional signal in glial cells in the fluorescence-based dataset. Overall, these analyses reveal diversity in the cellular distribution of ALS-RAP proteins. Notably, many ALS-associated proteins were detected across glial and immune cell populations, supporting the view that ALS genetics converges on multicellular disease mechanisms.

Overall, 16 of 27 proteins assessed at both the RNA and protein levels showed concordant cell type categorization (Figure 6B), highlighting both concordance and divergence between transcriptomic and protein-level datasets. Consistent with the limited protein-level characterization of ALS-associated genes, STRING network analysis revealed sparse evidence of direct interactions among the 33 proteins, with clusters primarily involving autophagy regulators (TBK1, OPTN, SQSTM1) and RNA-binding proteins (FUS, TARDBP, MATR3, HNRNPA1, HNRNPA2B1) (Figure 1—figure supplement 5). These clusters mainly comprise broadly expressed proteins, whereas interactions involving cell type-restricted ALS-RAP proteins remain largely unexplored.

## Discussion

Large-scale genetic studies have identified hundreds of risk loci across neurodegenerative diseases, including Alzheimer’s disease (Bellenguez et al., 2022; Zhang et al., 2023), Parkinson’s disease (Leonard and Global Parkinson’s Genetics Program (GP2), 2025; Kim et al., 2024), and ALS (van Rheenen et al., 2021). Despite these advances, functional follow-up has lagged behind genetic discovery, in part because disease-associated proteins lack validated reagents and systematic protein-level characterization. As a result, mechanistic insight has remained concentrated on a limited subset of genes, while the majority of risk-associated proteins remain poorly studied (Edwards et al., 2011; Carter et al., 2019). In ALS, this gap is particularly evident, with many proteins remaining part of the “dark ALS-ome”.

Here, we address this gap through ALS-RAP, which provides a publicly available dataset of KO-characterized antibodies across three commonly used applications—WB, IP, and IF—enabling the selection of fit-for-purpose reagents for studying ALS-associated proteins. Using these reagents, we examined protein levels across iPSC-derived and primary neurological cell types, revealing diverse expression patterns, including frequent detection of ALS-associated proteins in glial populations, particularly microglia and macrophages.

Consistent with this gap, STRING network analysis revealed sparse connectivity among ALS-associated proteins. Our expression analyses identify cell type-specific patterns that may help contextualize potential biological relationships. For example, ANXA11, classified as a definitive ALS gene by ClinGen, was predominantly detected in microglial populations. Annexins regulate membrane dynamics and trafficking, yet the role of Annexin A11 in microglia or macrophages remains largely unexplored. Its enrichment in microglia places it in the same cellular context as several ALS-associated proteins involved in autophagy and vesicular pathways, including C9orf72, TBK1, UBQLN2, SQSTM1, and OPTN, supporting potential convergence on shared pathways in myeloid-lineage cells. C9orf72 also showed higher expression in microglial populations, in agreement with studies implicating C9orf72 in microglial functions such as phagocytosis and immune regulation (O’Rourke et al., 2016; Masrori et al., 2025), as well as evidence that microglia present C9orf72-derived peptides capable of activating CD4^+^ T cells (Michaelis et al., 2025). Together, these findings support the view that ALS genetics converges on multicellular disease mechanisms involving both neuronal and glial populations.

## Materials and methods

**Key resources table:** 

Reagent type (species) or resource	Designation	Source or reference	Identifiers	Additional information
Antibody	HRP-Goat polyclonal anti-Rabbit antibody Secondary Antibody (H+L)	Thermo Fisher Scientific	CAT#65-6120; RRID:AB_2533967	0.05 µg/mL
Antibody	HRP-Goat polyclonal anti-Mouse antibody Secondary Antibody (H+L)	Thermo Fisher Scientific	CAT#62-6520; RRID:AB_2533947	0.1 µg/mL
Antibody	IRDye 680CW Goat polyclonal anti-Rabbit Secondary Antibody (H+L)	LI-COR Biosciences	CAT#926-68071; RRID:NA	0.05 µg/mL
Antibody	IRDye 680CW Goat polyclonal anti-Mouse Secondary Antibody (H+L)	LI-COR Biosciences	CAT#926-68070; RRID:NA	0.1 µg/mL
Antibody	IRDye 800CW Goat polyclonal anti-Rabbit Secondary Antibody (H+L)	LI-COR Biosciences	CAT#926-32211; RRID:NA	0.05 µg/mL
Antibody	IRDye 800CW Goat polyclonal anti-Mouse Secondary Antibody (H+L)	LI-COR Biosciences	CAT#926-32210; RRID:NA	0.1 µg/mL
Antibody	HRP-Goat multiclonal recombinant anti-Rabbit Secondary Antibody (H+L)	Proteintech	CAT#RGAR001; RRID:AB_3073505	0.05 µg/mL
Antibody	HRP-Goat multiclonal recombinant anti-Mouse Secondary Antibody (H+L)	Proteintech	CAT#RGAM001; RRID:AB_3068333	0.5 µg/mL
Antibody	CoraLite Plus 555-Goat multiclonal recombinant Anti-Rabbit Secondary Antibody (H+L)	Proteintech	CAT# RGAR003; RRID:AB_3073507	0.5 µg/mL
Antibody	CoraLite Plus 555-Goat multiclonal recombinant Anti-Mouse Secondary Antibody (H+L)	Proteintech	CAT# RGAM003; RRID:AB_3068539	0.5 µg/mL
Antibody	Rabbit recombinant monoclonal anti-Galectin-1 (clone EPR3205)	Abcam	CAT#ab108389; RRID:AB_10863417	WB:1/100; IP 2 µg; IF:1/100
Antibody	Rabbit recombinant monoclonal anti-Galectin-1 (clone EPR3206(2))	Abcam	CAT#ab138513; RRID:AB_2894851	WB:1/10,000; IP 2 µg; IF:1/250
Antibody	Rabbit recombinant monoclonal anti-Galectin-1 (clone SP247)	Abcam	CAT#ab192842; RRID:AB_3725515	WB:1/100; IP 2 µg; IF:1/100
Antibody	Rabbit recombinant monoclonal anti-Galectin-1 (clone ARC53156)	ABclonal	CAT#A21218; RRID:AB_3725523	WB:1/11,000; IP 2 µg; IF:1/500
Antibody	Rabbit polyclonal anti-Galectin-1	Aviva Systems Biology	CAT#ARP58491_P050; RRID:AB_2046045	WB:1/100; IP 2 µg; IF:1/250
Antibody	Mouse monoclonal anti-Galectin-1 (clone 933133)	Bio-Techne (R&D Systems)	CAT# MAB1152; RRID:AB_3657585	WB:1/250; IP 2 µg; IF:1/500
Antibody	Rabbit recombinant monoclonal anti-Galectin-1 (clone D608T)	Cell Signaling Technology	CAT#12936; RRID:AB_2798065	WB:1/1000; IP 2 µg; IF:1/50
Antibody	Rabbit recombinant monoclonal anti-Galectin-1 (clone JM13-37)	Thermo Fisher Scientific	CAT#MA5-32779; RRID:AB_2810056	WB:1/2000; IP 2 µg; IF:1/2000
Antibody	Rabbit recombinant monoclonal anti-Galectin-1 (clone A7W5Q6)	Thermo Fisher Scientific	CAT#MA5-35566; RRID:AB_2849466	WB:1/200; IP 2 µg; IF:1/100
Antibody	Rabbit recombinant monoclonal anti-Galectin-1 (clone SAIC-25B-112)	Thermo Fisher Scientific	CAT#MA5-47904; RRID:AB_3074348	WB:1/1000; IP 2 µg; IF:1/1000
Antibody	Rabbit recombinant monoclonal anti-CHMP2B (clone JE54-35)	Thermo Fisher Scientific	CAT#MA5-36184; RRID:AB_2890433	WB:1/500
Antibody	Rabbit recombinant monoclonal anti-FIG4 (clone N202/7R)	Addgene	CAT#192037; RRID:AB_2916241	WB:1/100
Antibody	Mouse monoclonal anti-FUS (clone CL0190)	Abcam	CAT#ab154141; RRID:AB_2885092	WB:1/1000
Antibody	Mouse monoclonal anti-HNRNPA1 (clone 4B10)	Bio-Techne (Novus Biologicals)	CAT#NB100-672; RRID:AB_524308	WB:1/1000
Antibody	Rabbit recombinant monoclonal anti-MATR3 (clone EPR10634(B))	Abcam	CAT#ab151739; RRID:AB_2885091	WB:1/10,000
Antibody	Rabbit polyclonal anti-NEK1	GeneTex	CAT#GTX130828; RRID:AB_2886357	WB:1/500
Antibody	Mouse monoclonal anti-OPTN (clone 6C1H4)	Proteintech	CAT#60293-1-Ig; RRID:AB_2881408	WB:1/5000
Antibody	Rabbit recombinant monoclonal anti-SIGMAR1 (clone D4J2E)	Cell Signaling Technology	CAT#61994; RRID:AB_2799617	WB:1/1000
Antibody	Mouse monoclonal anti-SOD1 (clone 2F10G1)	Proteintech	CAT#67480-1-Ig; RRID:AB_2882707	Chemiluminescent WB:1/10,000
Antibody	Rabbit polyclonal anti-SOD1	GeneTex	CAT#GTX100554; RRID:AB_10618670	Fluorescent WB:1/1000
Antibody	Rabbit recombinant monoclonal anti-SQSTM1 (clone EPR4844)	Abcam	CAT#ab109012; RRID:AB_2810880	Fluorescent WB:1/1000
Antibody	Mouse monoclonal anti-SQSTM1 (clone 2C11)	Abcam	CAT#ab56416; RRID:AB_945626	Chemiluminescent WB:1/1000
Antibody	Rabbit recombinant monoclonal anti-TAF15 (clone JE61-92)	Thermo Fisher Scientific	CAT#MA5-44822; RRID:AB_2931279	WB:1/1000
Antibody	Rabbit recombinant monoclonal anti-TARDBP (clone 11N20)	Proteintech	CAT#80001-1-RR; RRID:AB_2882933	WB:1/10,000
Antibody	Rabbit recombinant monoclonal anti-TBK1 (clone EP611Y)	Abcam	CAT#ab40676; RRID:AB_776632	Chemiluminescent WB:1/10,000
Antibody	Rabbit recombinant monoclonal anti-TBK1 (clone E8I3G)	Cell Signaling Technology	CAT#38066; RRID:AB_2827657	Fluorescent WB:1/1000
Antibody	Rabbit recombinant monoclonal anti-TIA1 (clone D1Q3K)	Cell Signaling Technology	CAT#86050; RRID:AB_2800070	WB:1/1000
Antibody	Mouse monoclonal anti-UBQLN2 (clone 6H9)	Abcam	CAT#ab190283; RRID:AB_2747782	WB:1/1000
Antibody	Rabbit recombinant monoclonal anti-VCP (clone 7F3)	Cell Signaling Technology	CAT#2649; RRID:AB_2214629	Chemiluminescent WB:1/10,000
Antibody	Rabbit recombinant monoclonal anti-VCP (clone EPR3307(2))	Abcam	CAT#ab109240; RRID:AB_10862588	Fluorescent WB:1/500
Antibody	Rabbit polyclonal anti-ANXA11	Aviva Systems Biology	CAT#ARP87950_P050; RRID:AB_2894925	WB:1/1000
Antibody	Mouse monoclonal anti-C9orf72 (clone GT1553)	GeneTex	CAT#GTX634482; RRID:AB_2784545	WB:1/1000
Antibody	Rabbit polyclonal anti-CHCHD10	Proteintech	CAT#25671-1-AP; RRID:AB_2880187	Chemiluminescent WB:1/500
Antibody	Mouse monoclonal anti-CHCHD10 (clone OTI3B8)	Thermo Fisher Scientific	CAT#MA5-27535; RRID:AB_2724132	Fluorescent WB:1/500
Antibody	Rabbit recombinant monoclonal anti-LGALS1 (clone EPR3206(2))	Abcam	CAT#ab138513; RRID:AB_2894851	WB:1/500
Antibody	Rabbit recombinant monoclonal anti-PFN1 (clone ARC1469)	ABclonal	CAT#A9188; RRID:AB_2863682	WB:1/10,000
Antibody	Rabbit recombinant monoclonal anti-SPG11 (clone 2H25L11)	Thermo Fisher Scientific	CAT#702776; RRID:AB_2725406	WB:1/200
Antibody	Mouse monoclonal anti-ACSL5 (clone 5H8)	Abnova	CAT#H00051703-M01; RRID:AB_509241	WB:1/200
Antibody	Rabbit recombinant monoclonal anti-CAV1 (clone SZ02-01)	Thermo Fisher Scientific	CAT#MA5-32020; RRID:AB_2809314	Chemiluminescent WB:1/500
Antibody	Rabbit recombinant monoclonal anti-CAV1 (clone ARC50848)	ABclonal	CAT#A19006; RRID:AB_2862498	Fluorescent WB:1/500
Antibody	Rabbit recombinant monoclonal anti-ATXN2 (clone EPR23630-49)	Abcam	CAT#ab254362; RRID:AB_2885130	WB:1/1000
Antibody	Rabbit recombinant monoclonal anti-TUBA4A (clone EPR13478(B))	Abcam	CAT#ab176560; RRID:AB_2860019	Chemiluminescent WB:1/500
Antibody	Rabbit recombinant monoclonal anti-TUBA4A (clone JM73-24)	Thermo Fisher Scientific	CAT#MA5-32738; RRID:AB_2810015	Fluorescent WB:1/1000
Antibody	Rabbit recombinant monoclonal anti-VAPB (clone EPR27026-50)	Abcam	CAT#ab315013; RRID:AB_3086770	Chemiluminescent WB:1/500
Antibody	Rabbit polyclonal anti-VAPB	Thermo Fisher Scientific	CAT#PA5-53023; RRID:AB_2649391	Fluorescent WB:1/200
Antibody	Rabbit recombinant monoclonal anti-ALS2 (clone EPR11185)	Abcam	CAT#ab170896; RRID:AB_2885099	WB:1/1000
Antibody	Mouse monoclonal anti-HNRNPA2B1 (clone DP3B3)	Bio-Techne (Novus Biologicals)	CAT#NB120-6102; RRID:AB_790226	WB:1/500
Antibody	Rabbit recombinant monoclonal anti-KIF5A (clone 241124H1)	Proteintech	CAT#84013-4-RR; RRID:AB_3671581	WB:1/500
Antibody	Rabbit recombinant monoclonal anti-Synaptotagmin 1 (clone HL1626)	GeneTex	CAT#GTX637119; RRID:AB_3068350	WB:1/500
Antibody	Rabbit recombinant monoclonal anti-CD44 (clone EPR18668)	Abcam	CAT#ab189524; RRID:AB_2885107	WB:1/1000
Antibody	Rabbit recombinant monoclonal anti-ISL1 (clone EPR10362)	Abcam	CAT#ab178400; RRID:AB_2927537	WB:1/500
Antibody	Rat recombinant monoclonal anti-Myelin Basic Protein antibody (clone 12)	Abcam	CAT#ab7349; RRID:AB_305869	WB:1/500
Antibody	Rabbit polyclonal anti-Tyrosine Hydroxylase	Pel-Freeze	CAT#P40101-150: RRID:AB_2617184	WB:1/500
Antibody	Rabbit recombinant monoclonal anti-CD11b (clone EPR1344)	Abcam	CAT#ab133357; RRID:AB_2650514	WB:1/500
Antibody	Rabbit recombinant monoclonal anti-IBA1 (clone E4O4W)	Cell Signaling Technology	CAT#17198; RRID:AB_2820254	WB:1/500
Antibody	Chicken monoclonal recombinant anti-IBA1 (clone Ch311H9)	Synaptic Systems	CAT#234009; RRID:AB_2891282	IF:1/1000
Antibody	Mouse monoclonal anti-CD11b, pre-conjugated with PE (clone ICRF44)	BD Biosciences	CAT#555388; RRID:AB_395789	IF:1/20
Antibody	Mouse monoclonal anti-CD45, pre-conjugated with Alexa Fluor 700 (clone HI30)	BioLegend	CAT#304024; RRID:AB_493761	IF:1/40
Antibody	Rabbit polyclonal anti-PU.1	Cell Signaling Technology	CAT#2266; RRID:AB_10692379	IF:1/500
Commercial assay or kit	RNeasy Mini Kit	QIAGEN	CAT#74106	
Commercial assay or kit	STEMdiff Forebrain Neuron Differentiation Kit	STEMCELL Technologies	CAT#08600	
Commercial assay or kit	STEMdiff Forebrain Neuron Maturation Kit	STEMCELL Technologies	CAT#08605	
Commercial assay or kit	STEMdiff Hematopoietic Kit	STEMCELL Technologies	CAT#05310	
Commercial assay or kit	STEMdiff Hematopoietic Kit	STEMCELL Technologies	CAT#05310	
Commercial assay or kit	STEMdiff Hematopoietic Kit	STEMCELL Technologies	CAT#05310	
Cell line (human)	HAP1 WT	Horizon Discovery	CAT#C631; RRID:CVCL_Y019	Cells were confirmed mycoplasma-free
Cell line (human)	HAP1 *LGALS1* KO	Horizon Discovery	CAT#HZGHC005989c006; RRID:CVCL_XQ14	Cells were confirmed mycoplasma-free
Cell line (human)	Healthy control iPSCs from the AIW002-02 background	EDDU, McGill University	NA	Cells were confirmed mycoplasma-free
Cell line (human)	Healthy control iPSCs from the QPN929 background	EDDU, McGill University	NA	Cells were confirmed mycoplasma-free
Software, algorithm	LI-COR Image Studio Lite	licorbio.com/image-studio	NA	
Software, algorithm	FIJI Software	fiji.sc/	RRID:SCR_002285	
Software, algorithm	MetaXpress High-Content Screening Software version 6.7.1.157	moleculardevices.com/products/cellular-imaging-systems/high-content-analysis/metaxpress - overview	RRID:SCR_016654	
Software, algorithm	PyCharm 2023.3.4	jetbrains.com/pycharm/	RRID:SCR_018221	
Software, algorithm	RStudio 2023.09.1	cran.rstudio.com/	RRID:SCR_000432	
Other	Accutase	STEMCELL Technologies	CAT#07922	
Other	Antibody Registry	antibodyregistry.org	RRID:SCR_006397	
Other	Astrocyte Growth Supplements (AGS)	ScienCell Research Laboratories	CAT#1852	
Other	Astrocyte Medium (AM)	ScienCell Research Laboratories	CAT#1801-b	
Other	BLUelf Prestained Protein Ladder	FroggaBio	CAT#PM008	
Other	Cellosaurus—- a knowledge resource on cell lines	cellosaurus.org	RRID:SCR_013869	
Other	CellTracker deep red dye	Thermo Fisher Scientific	CAT#C34565	
Other	CellTracker green chloromethyl derivative of fluorescein diacetate (CMFDA) dye	Thermo Fisher Scientific	CAT#C2925	
Other	Centrifugal filter unit, Amicon Ultra-15	MilliporeSigma	CAT#UFC901096	
Other	CiteAb—Reagent Search & Life Science Data	citeab.com	RRID:SCR_009653	
Other	ClinGen	https://clinicalgenome.org/	RRID:SCR_014968	
Other	DepMap: The Cancer Dependency Map Project at Broad Institute	depmap.org/portal	RRID:SCR_017655	
Other	Live/Dead Fixable Aqua viability dye	Thermo Fisher Scientific	CAT#L34965	
Other	mTeSR1	STEMCELL Technologies	CAT#85850	
Other	NuPAGE Bis-Tris Midi Protein Gels, 10%, 1.0 mm	Thermo Fisher Scientific	CAT#WG1201BOX	
Other	NuPAGE MES SDS Running Buffer (20×)	Thermo Fisher Scientific	CAT#NP000202	
Other	NuPAGE MOPS SDS Running Buffer (20×)	Thermo Fisher Scientific	CAT#NP000102	
Other	PhenoPlate, 96-well, optically clear flat bottom	PerkinElmer	CAT#6055300	
Other	Pierce IP Lysis Buffer	Thermo Fisher Scientific	CAT#87788	
Other	PubMed	pubmed.ncbi.nlm.nih.gov	RRID:SCR_004846	
Other	RIPA Lysis and Extraction Buffer	Thermo Fisher Scientific	CAT#89901	
Other	Structural Genomics Consortium	https://www.thesgc.org/	RRID:SCR_003890	
Other	The Human Protein Atlas	proteinatlas.org	RRID:SCR_006710	
Other	TRIzol	Thermo Fisher Scientific	CAT#15596026	
Other	TrueStain blocking reagent	BioLegend	CAT#422302	
Other	Zenodo	https://zenodo.org/	RRID:SCR_004129	

### Analysis of PubMed entries for each ALS-associated gene

Throughout the manuscript, we reference ALS-RAP targets using their official gene names as listed in UniProt, written in italics (e.g., *SOD1*). To refer to the corresponding proteins, we use the same gene names in regular font, as the official protein names are often too long or ambiguous for practical use.

Each target gene was queried in PubMed using two search strategies: (1) GENE_NAME[Title/Abstract] to retrieve the total number of publications associated with the gene, and (2) (GENE_NAME[Title/Abstract]) AND (“amyotrophic lateral sclerosis” [Title/Abstract]) to identify publications specifically linking the gene to ALS. Searches were performed on August 27, 2025.

### Evaluation of significance of ALS-associated genes

Each candidate gene was assessed for its potential association with ALS using the evidence published at the time of this article. Using the recent review of Nijs and van Damme as a model (Nijs and Van Damme, 2024), we used currently available evidence to prioritize genes for potential association with ALS in a similar manner. First, genes were qualitatively assessed for rarity of genetic variants in the general population and among individuals with ALS. Next, any reported odds ratios (ORs) for risk of ALS in variant carriers were used to approximate whether genes were considered to have “small” (OR <1.2), “intermediate” (1.2<OR < 10), or “high” (OR >10) effect sizes. Genes were assigned an “uncertain” OR estimate if no OR had been published at the time of analysis. Variant frequency was assessed considering the type(s) of inheritance previously described; variants reported through GWAS or genotyping studies were designated as “common”, whereas variants reported in autosomal family/pedigree-based studies were designated as “rare”. If the method of discovery was poorly described or through non-genetic analyses (e.g., transcriptomic or proteomic), genes were described as having “uncertain” frequency.

### RNA expression analysis

We accessed the single-cell transcriptomics resource of the HPA, specifically the single-nuclei brain dataset that includes single-cell RNA sequencing data generated from 2.5 million cells and mapped to 34 supercluster cell types. Data were generated from postmortem human brain samples digested to a single-cell suspension, and cell types were clustered into transcriptomic cell types (Siletti et al., 2023). Raw sequencing data was normalized using Trimmed mean of M values and expression values were presented as normalized transcripts per million (nTPM). RNA expression data for all 33 ALS genes were downloaded online from the HPA. Data were grouped together based on overall cell type (neuron, oligodendrocyte, astrocyte, microglia, etc.), and the average expression in each cell type was determined for each gene. The average neuron, oligodendrocyte, astrocyte, and microglia expression data were extracted from the overall dataset and used in downstream analyses.

In our analyses, genes were considered “expressed” if their expression values in at least three cell types were greater than the median expression value of the entire HPA dataset (9.2 nTPM). Genes were considered “barely detectable” if expression was less than the median value in at least two cell types. For a small subset of “barely detectable” genes, expression in one cell type was greater than the median value and substantially “enriched” in that cell type. We included these in the category of “expressed” genes despite the low expression in most cell types.

To describe the cell type expression patterns for each gene, we established criteria determining if mRNA expression levels for a gene is “enriched” or not in a given cell type. Cell types in which mRNA expression for a gene is “enriched” were defined as when the ratio of the expression value in that cell type to the average expression value across all cell types was greater than or equal to 1.5*. Each gene was categorized based on the “enriched” cell types. If either more than one cell type was “enriched” or no cell types were, the gene was categorized as having “low cell-type specificity”.

$$\displaystyle \frac{* Expression\ value\ in\ given\ cell\ type}{Average\ across\ all\ cell\ types}\geq 1.5$$

### STRING analysis

STRING analysis was performed by entering the UniProt IDs of the 33 ALS-RAP proteins into the “multiple proteins” search at string-db.org. The analysis was restricted to the “physical subnetwork”, with the meaning of network edges set at “confidence”, and the minimum required interaction score set to “highest confidence”.

### Western blot (WB)

Primary cells, iPSC-derived cultures and cultured cells were collected in RIPA buffer (25 mM Tris-HCl pH 7.6, 150 mM NaCl, 1 % NP-40, 1% sodium deoxycholate, 0.1% SDS) supplemented with protease inhibitors. Lysates were sonicated briefly for 5–10 seconds depending on the volume. Following 30 minutes on ice, lysates were spun at ~110,000 × *g* for 15 minutes at 4°C and equal protein aliquots of the supernatants were analyzed by SDS-PAGE and WB.

#### Chemiluminescent WB

WB were performed with precast 10% Bis-Tris polyacrylamide gels (MOPS or MES SDS buffer) and nitrocellulose membranes. Total proteins were visualized with a Ponceau staining. Blots were blocked with 5% milk in TBS with 0.1% Tween 20 (TBST). Antibodies were also diluted in 5% milk in TBST and incubated O/N at 4°C. Membranes were washed three washes in TBST the next day. The peroxidase conjugated anti-rabbit and anti-mouse secondary antibodies (Thermo Fisher Scientific) were diluted in 5% milk in TBST at 0.5 and 1 µg/mL, respectively. Secondaries were incubated with the membranes for 1 hour at room temperature followed by three washes. Membranes were incubated with ECL for 1 minute prior to detection with the iBright CL1500 Imaging System (Thermo Fisher Scientific).

#### Fluorescent WB

WB were performed with large 5–16% gradient Tris-based polyacrylamide gels (Tris-Glycine/SDS Buffer) and nitrocellulose membranes. Total proteins were visualized with the REVERT total protein stain (LI-COR Biosciences). Blots were blocked with 5% milk in TBST, and antibodies were diluted in the same buffer and incubated O/N at 4°C followed by three washes. The secondary antibodies (Odyssey IRDye 800CW or IRDye 680RD) were diluted in 5% milk in TBST to a final concentration of 0.05 µg/mL for anti-rabbit and 0.1 µg/mL for anti-mouse. Secondaries were incubated with the membranes for 1 hour at room temperature followed by three washes while covered from the light. Immuno-reactive bands were detected using the LI-COR Odyssey Imaging System (LI-COR Biosciences), and data analysis was done using LI-COR Image Studio Lite Version 6.10.2.

### Galectin-1 antibody screening in WB

For cell lysis, HAP1 WT and *LGALS1* KO cells were collected in RIPA buffer and processed as described above.

To collect the conditioned medium, HAP1 WT and *LGALS1* KO cells were washed 3× with PBS 1× and starved for ~36 hours in Iscove’s Modified Dulbecco’s Medium supplemented with 1% Pen/Strep and 1% L-Glutamine. Culture media were collected and centrifuged for 10 minute at 500 × *g* to eliminate cells and larger contaminants, then for 10 minutes at 4500 × *g* to eliminate smaller contaminants. Culture media were then concentrated by centrifuging at 4000 × *g* for 30 minutes using the Amicon Ultra-15 Centrifugal Filter Units with a membrane NMWL of 10 kDa. Culture media were finally supplemented with 1× protease inhibitor cocktail mix.

10% Bis-Tris gels with MES buffer were used for SDS-PAGE and WB was performed using the primary antibodies (Table 2) at the following dilutions: ab108389 at 1/100, ab138513 at 1/10,000, ab192842 at 1/100, A21218 at 1/11,000, ARP58491 at 1/100, MAB1152 at 1/250, 12936 at 1/1000, MA5-32779 at 1/2000, MA5-35566 at 1/200, and MA5-47904 at 1/1000. Anti-rabbit and anti-mouse antibodies (Proteintech) were used at a final concentration of 0.05 µg/mL and 0.5 µg/mL, respectively.

### Galectin-1 antibody screening in immunoprecipitation (IP)

Antibody-bead conjugates were prepared by adding 2 µg to 500 µL of Pierce IP Lysis Buffer from Thermo Fisher Scientific in a microcentrifuge tube, together with 30 µL of Dynabeads protein A- (for rabbit antibodies). Tubes were rocked for 1 hour at 4°C followed by two washes to remove unbound antibodies. HAP1 WT were collected in Pierce IP buffer (25 mM Tris-HCl pH 7.4, 150 mM NaCl, 1 mM EDTA, 1% NP-40, and 5% glycerol) supplemented with protease inhibitor. Lysates were rocked 30 minutes at 4°C and spun at 110,000 × *g* for 15 minutes at 4°C. 0.2 mL aliquots at 1 mg/mL of lysate were incubated with each antibody-bead conjugate for 2 hour at 4°C. To run 4% SM (8 µL per SM) alongside each IP on the gel, a master mix of lysate was kept aside on ice for subsequent SM sample preparation. The UBs were collected separately and prepared for gel loading similar to the rest of the samples. Beads were washed three times with 1.0 mL of IP buffer and processed for SDS-PAGE on precast midi 10% Bis-Tris polyacrylamide gels. For WB, membranes were trimmed at 24 kDa and the lower part was incubated with the rabbit recombinant monoclonal anti-Galectin-1 antibody A21218 diluted at 1/3000 in 5% milk in TBST. HRP-conjugated anti-rabbit (Proteintech) diluted in 5% milk in TBST was used as a secondary antibody at 0.05 µg/mL.

### Galectin-1 antibody screening in immunofluorescence (IF)

WT and *LGALS1* KO HAP1 cells were labeled with a green and a far-red fluorescence dye, respectively. WT and KO cells were plated in a 96-well plate with optically clear flat-bottom (PerkinElmer) as a mosaic and incubated for 24 hours in a cell culture incubator at 37°C, 5% CO_2_. Cells were fixed in 4% paraformaldehyde (PFA) in phosphate-buffered saline (PBS). Cells were permeabilized in PBS with 0.1% Triton X-100 for 10 minutes at room temperature and blocked with PBS with 5% BSA, 5% goat serum, and 0.01% Triton X-100 for 30 minutes at room temperature. Cells were incubated with IF buffer (PBS, 5% BSA, 0.01% Triton X-100) containing the primary Galectin-1 antibodies overnight at 4°C. Cells were then washed 3×10 minutes with IF buffer and incubated with the corresponding CoraLite Plus 555 secondary antibody in IF buffer at a dilution of 0.5 µg/mL for 1 hour at room temperature. Cells were washed 3×10 minutes with 1× PBS. The nuclei were labeled with DAPI fluorescent stain at a concentration of 5 ng/mL in 1× PBS for 3 minutes then washed once with 1× PBS.

Images were acquired on an ImageXpress micro confocal high-content microscopy system (Molecular Devices). The objective used is a water Apo LambdaS LWD with magnification of 20×, NA 0.95, and scientific CMOS cameras, equipped with 395, 475, 555, and 635 nm solid-state LED lights (lumencor Aura III light engine) and bandpass filters to excite DAPI, Cellmask Green, Coralite-555, and Cellmask Red, respectively. Images had pixel sizes of 0.68 × 0.68 microns, and a z-interval of 4 microns. For analysis and visualization, shading correction (shade only) was carried out for all images. Then, maximum intensity projections were generated using three z-slices. Segmentation was carried out separately on maximum intensity projections of Cellmask channels using CellPose 1.0, and masks were used to generate outlines and for intensity quantification.

### iPSC differentiation protocols

Healthy control iPSCs from the AIW002-02 and QPN929 backgrounds (Early Drug Discovery Unit-EDDU, McGill University) were selected for differentiation into the indicated neurological cell types. In all cases, iPSCs were cultured in mTeSR1 for 5–7 days prior to differentiation. Two batches of neurological cells were generated from the AIW002-02 background using identical protocols, with the exception that the QPN929 iPSC line was used for microglia differentiation in the batch used for chemiluminescent WB.

### Motor neuron differentiation

Motor neurons were differentiated from iPSCs following a previously published protocol (Deneault et al., 2022; Lépine et al., 2024). Basic neural medium (BNM, 1:1 mixture of DMEM/F12 and Neurobasal, 0.5X N2, 0.5X B27, 0.5X GlutaMAX, 1X antibiotic-antimycotic, and 100 μM ascorbic acid) serves as the base medium for all other formulations used in this protocol. iPSCs were seeded on matrigel-coated flasks in neural induction medium (BNM supplemented with 2 μM SB431542, 2 μM DMH1, 3 μM CHIR99021) with a full media change performed every other day. After 6 days, cells were passaged in patterning medium (BNM supplemented with 1 μM CHIR99021, 2 μM SB431542, 2 μM DMH1, 0.1 μM retinoic acid [RA] and 0.5 μM purmorphamine [PMN]) onto poly-L-ornithine- and laminin-coated flasks and cultured for a further 6 days with a full media change performed every other day. Cells were then passaged onto poly-L-ornithine- and laminin-coated flasks in expansion medium (BNM supplemented with 3 μM CHIR99021, 2 μM SB431542, 2 μM DMH1, 0.1 μM RA, and 0.5 μM PMN, and 0.5 mM valproic acid [VPA]), with a full media change performed every other day for 6 days to obtain motor neural progenitor cells (M-NPCs). M-NPCs were dissociated as single cells and seeded onto poly-L-ornithine- and laminin-coated plates in final differentiation medium (BNM supplemented with 0.5 μM RA, 0.1 μM PMN, 0.1 μM Compound E [CpdE], 10 ng/mL insulin-like growth factor-1 [IGF-1], brain-derived neurotrophic factor [BDNF], and ciliary neurotrophic factor [CNTF]), with a half media change once per week for final differentiation into motor neurons (MNs). MNs were considered mature and collected for WB after 4 weeks of differentiation.

### Dopaminergic neuron differentiation

Dopaminergic neurons were differentiated from iPSCs following a protocol of floor-plate induction with additional modifications (Kriks et al., 2011; Chen et al., 2022). Briefly, iPSCs were dissociated into single cells to form embryoid bodies (EBs). EBs were grown on low-attachment plates for 1 week in DMEM/F12 supplemented with 1X N2 and 1X B27, in the presence of 10 μM SB431542, 200 ng/mL Noggin, 200 ng/mL sonic hedgehog, 3 μM CHIR99021, and 100 ng/mL Fibroblast growth factor 8 (FGF8). On day 7, EBs were transferred, in the same media, to poly-L-ornithine- and laminin-coated plates. On day 14, rosettes were selected semi–manually and cultured as a monolayer in DMEM/F12 supplemented with 1X N2 and 1X B27 on poly-L-ornithine- and laminin-coated plates to generate dopaminergic neural progenitor cells (DA-NPCs). DA-NPCs were passaged at a ratio of 1:3 every 5–7 days. DA-NPCs were then cultured in neurobasal medium, supplemented with 1X N2 and 1X B27, in the presence of 1 μg/mL laminin, 500 μM dibutyryl-cAMP (db-cAMP), 20 ng/mL BDNF, 20 ng/mL glial-derived neurotrophic factor (GDNF), 200 μM ascorbic acid, 100 nM CpdE, and 1 ng/mL transforming growth factor β3 (TGF-β3), with a half media change twice per week for final differentiation into dopaminergic neurons. Dopaminergic neurons were considered mature and collected for WB after 4 weeks of differentiation.

### Oligodendrocyte differentiation

Oligodendrocytes were differentiated from iPSCs following a recently published protocol (Piscopo et al., 2024). The base media for all other formulations in this protocol is as follows: DMEM/F12 with GlutaMAX supplemented with 1X N2, 1X B27 without vitamin A, 1X NEAA, 55 mM β-mercaptoethanol, 25 mg/mL Insulin, and 2 mg/mL heparin. First, iPSCs were directed toward a neural progenitor fate in base media supplemented with 10 µM SB31542 and 2 µM DMH1, with daily media change over 8 days. Followed by patterning with 100 nM RA and 1 µg/mL PMN in base media with daily media change, for 4 days. Cells were then transferred from monolayer to suspension culture in the same medium for an additional 8 days to form neurospheres. Oligodendrocyte precursor cells were then generated and expanded in suspension using OPC media (base media supplemented with 10 ng/mL PDGF-AA, 10 ng/mL IGF-1, 10 ng/mL NT-3, 5 ng/mL HGF, 60 ng/mL 3, 3′, 5-Triiodo-L-thyronine [T3], 100 ng/mL biotin, and 1 µM db-cAMP) for 10 days. Following this, the cells were plated in OPC media onto poly-L-ornithine/laminin-coated vessels with half media changes performed every other day to support further differentiation toward the OPC lineage, which continues until day 75. At this stage, cells were detached and passaged using TrypLE Express Enzyme. In the final differentiation step, cells were exposed to ScienCell Astrocyte Medium supplemented with ScienCell Astrocyte Growth Supplements (AGS) for 10 days. AGS was then withdrawn, and final maturation completed by reintroducing IGF-1 and T3 at the previously described concentrations for a further 10 days, with half media changes performed every other day. At this point, oligodendrocytes were considered mature and collected for WB.

### Astrocyte differentiation

Astrocytes were differentiated from iPSCs following a previously published protocol (Soubannier et al., 2022). First, iPSCs were differentiated into neural progenitor cells following the steps described above for the generation of M-NPCs. M-NPCs were dissociated as single cells and plated at a low cell density (15,000 cells/cm^2^) onto a matrigel-coated vessel in M-NPC expansion media (as described above). The next day, medium was replaced with Astrocyte Differentiation Medium (ScienCell Astrocyte Medium containing AGS, 1% fetal bovine serum [FBS] [ScienCell Research Laboratories], 1% penicillin-streptomycin [Wisent Bioproducts]). Cells were passaged in the same media at a ratio of 1:2 once per week and were maintained under these culture conditions for 30 days, with a half media change performed every 3–4 days. FBS was then withdrawn to promote astrocyte maturation. Cells were maintained for an additional 4 weeks with a half media change performed twice per week prior to astrocyte collection for WB.

### Microglia differentiation

iPSC-derived microglia were differentiated following a published protocol (McQuade et al., 2018). Briefly, iPSCs were differentiated to hematopoietic precursor cells (iHPCs) using the STEMCELL Technologies STEMdiff Hematopoietic Kit. iHPCs were harvested on days 10 and 12 of iHPC differentiation and seeded on matrigel-coated 6-well plates in microglia differentiation media (MDM, DMEM/F12 HEPES no phenol red supplemented with 2X insulin-transferrin-selenite, 2X B27, 0.5X N2, 1X GlutaMAX, 1X NEAA, 400 µM monothioglycerol, and 5 µg/mL insulin)+three cytokine cocktail (25 ng/mL M-CSF, 100 ng/mL IL-34, and 50 ng/mL TGF-β1) for further differentiation into microglia. Cells were supplemented with MDM +three cytokine cocktail every other day, with a full media change performed on day 12, for 24 days of microglia differentiation. On day 25, a full media change to MDM + five cytokine cocktail (25 ng/mL M-CSF, 100 ng/mL IL-34, 50 ng/mL TGF-β1, 100 ng/mL CD200, and 100 ng/mL CX3CL1) was performed, followed by supplementation with MDM + five cytokine cocktail on day 27. Microglia were considered mature and utilized for downstream experiments after 28 days of microglial differentiation.

### Flow cytometry analysis of CD45 and CD11b

Mature iPSC-derived microglia were collected by combining non-adherent cells recovered from culture media with adherent cells gently detached in PBS. Undifferentiated iPSCs were dissociated into single-cell suspensions using Accutase. Cells were first incubated with Live/Dead Fixable Aqua viability dye in PBS for 30 minutes at room temperature, followed by washing in FACS buffer (PBS supplemented with 1% FBS and 0.1% sodium azide). Fc receptor blocking was performed using TrueStain blocking reagent for 10 minutes prior to antibody staining.

Cells were then incubated with fluorophore-conjugated antibodies against CD11b (PE; 1:20) and CD45 (Alexa Fluor 700; 1:40) for 15 minutes at room temperature. After washing, cells were resuspended in FACS buffer and analyzed using a Thermo Attune NxT flow cytometer equipped with 405, 488, and 561 nm lasers (NeuroEDDU Flow Cytometry Facility, McGill University). A minimum of 50,000 events per sample were acquired. Analysis was performed in FlowJo (BD Biosciences), with sequential gating applied to exclude debris and doublets, followed by selection of live cells prior to assessment of CD45 and CD11b expression.

### Immunofluorescence staining for IBA1 and PU.1

Mature iPSC-derived microglia were plated onto glass coverslips and fixed in 4% paraformaldehyde for 20 minutes at room temperature. Following three washes in PBS, cells were permeabilized with 0.2% Triton X-100 in PBS for 10 minutes and blocked in 5% normal donkey serum for 1 hour. Primary antibodies against IBA1 (Synaptic Systems 234 009, 1:1,000) and PU.1 (Cell Signaling Technology 2266, 1:500) were diluted in blocking solution and incubated overnight at 4°C. After washing in PBS, cells were incubated for 1 hour at room temperature with corresponding secondary antibodies, together with Hoechst (1 μg/mL). Coverslips were washed, mounted, and imaged using a Leica SP8 confocal microscope.

### RNA extraction and quantitative RT-PCR

Total RNA was isolated using TRIzol reagent followed by purification with the RNeasy Mini Kit (QIAGEN 74106) according to the manufacturer’s instructions. RNA concentration and purity were assessed by spectrophotometry. Reverse transcription was performed using 500 ng total RNA with M-MLV reverse transcriptase, random hexamers, RNase inhibitor, and dNTPs in first-strand buffer supplemented with DTT. Reactions were incubated at 42°C for 60 minutes followed by enzyme inactivation at 75°C for 10 minutes.

Quantitative PCR was performed using TaqMan gene expression assays (Thermo Fisher) on a QuantStudio 3 Real-Time PCR System. Relative expression levels were calculated using the comparative Ct (ΔΔCt) method with GAPDH as the reference gene.

The following TaqMan probes were used: GAPDH–Hs02786624_g1; AIF1 (IBA1)–Hs00610419_g1; OLFML3–Hs01113293_g1; SPI1 (PU.1)–Hs02786711_m1; CX3CR1–Hs01922583_s1; MERTK–Hs01031969_m1; TREM2–Hs00219132_m1; CSF1–Hs00174164_m1; P2RY12–Hs01881698_s1; RUNX1–Hs02558380_s1.

## Data Availability

All data generated during this study are publicly available through the YCharOS community on Zenodo (https://zenodo.org/communities/ycharos/). This includes raw datasets from antibody characterization studies for 33 ALS-associated proteins, as well as raw immunoblot data used for protein expression profiling. Data are organized as individual records corresponding to each antibody characterization study or immunoblot experiment. DOIs for antibody characterization datasets are provided in Table S1, and DOIs for expression profiling datasets are cited in the manuscript. The following datasets were generated: MoleónVR FantiR FotouhiM AlendeC RyanJ AyoubiR DurcanTM BrownCM McPhersonPS LaflammeC 2024Antibody Characterization Report for ACSL5 (Long-chain-fatty-acid--CoA ligase 5)Zenodo10.5281/zenodo.10891256 MoleónVR BolívarSG AyoubiR McPhersonPS LaflammeC NeuroSGC/YCharOS/EDDU collaborative group ABIF consortium 2025Antibody Characterization Report for AlsinZenodo10.5281/zenodo.17953903 AyoubiR WorrallD McPhersonPS LaflammeC 2023Antibody Characterization Report for AngiogeninZenodo10.5281/zenodo.7671286 AlshafieW AyoubiR NicouleauM DurcanTM McPhersonPS LaflammeC 2022Antibody Characterization Report for Annexin A11Zenodo10.5281/zenodo.5903684 AlshafieW FotouhiM SouthernK McPhersonPS LaflammeC 2021Antibody Characterization Report for Ataxin-2Zenodo10.5281/zenodo.5061824 AyoubiR BolívarSG McPhersonPS LaflammeC 2025A guide to selecting high-performing antibodies for Caveolin-1 (UniProt ID: Q03135) for use in western blotZenodo10.5281/zenodo.17253403 MoleónVR AyoubiR McPhersonPS LaflammeC 2023Antibody Characterization Report for Cyclin-FZenodo10.5281/zenodo.7971946 AyoubiR AlshafieW SouthernK McPhersonPS LaflammeC NeuroSGC/YCharOS/EDDU collaborative group 2023The identification of high-performing antibodies for Coiled-coil-helix-coiled-coil-helix domain containing protein 10 (CHCHD10) for use in Western Blot, immunoprecipitation and immunofluorescenceF1000Research10.12688/f1000research.133479.2PMC1052110037767023 AlshafieW FotouhiM AyoubiR ShlaiferI SouthernK McPhersonPS LaflammeC NeuroSGC/YCharOS/EDDU collaborative group 2023The identification of high-performing antibodies for Charged multivesicular body protein 2b for use in Western Blot, immunoprecipitation and immunofluorescenceF1000Research10.12688/f1000research.139755.1PMC1044814437635943 MoleónVR FotouhiM AlendeC RyanJ AyoubiR ReintschW DurcanTM BrownCM McPhersonPS LaflammeC 2024Antibody Characterization Report for FIG4Zenodo10.5281/zenodo.10814961 AlshalfieW BiddleM FotouhiM JonesC AyoubiR YouZ SouthernK McPhersonPS VirkH LaflammeC NeuroSGC/YCharOS/EDDU collaborative group F1000Research2024The identification of high-performing antibodies for FUS (UniProt ID: P35637) for use in western blot, immunoprecipitation, immunofluorescence and flow cytometry10.12688/f1000research.133220.3PMC1029379937384305 MoleónVR FotouhiM AlendeC RyanJ AyoubiR ReintschW DurcanTM BrownCM McPhersonPS LaflammeC 2024Antibody Characterization Report for hnRNP A1 (Heterogeneous nuclear ribonucleoprotein A1)Zenodo10.5281/zenodo.10815038 MoleónVR AyoubiR AlendeC FothouhiM RyanJ BolívarSG WorrallD DurcanTM BrownCM FrancisV McPhersonPS LaflammeC 2025A guide to selecting high-performing antibodies for Heterogeneous nuclear ribonucleoproteins A2/B1 (hnRNP A2/B1) (UniProt ID: P22626) for use in western blot, immunoprecipitation, and immunofluorescenceF1000Research10.12688/f1000research.170886.1PMC1266997641341144 AyoubiR BolívarSG LaflammeC 2026A guide to selecting high-performing antibodies for KIF5A (Q12840) for use in western blotZenodo10.5281/zenodo.18612357PMC1356301742732114 ZhaCY AyoubiR AlendeC FotouhiM BolívarSG LaflammeC 2026A guide to selecting high-performing antibodies for Galectin-1 (P09382) for use in western blot, immunoprecipitation and immunofluorescenceZenodo10.5281/zenodo.18612277 AyoubiR FotouhiM AlendeC RyanJ ReintschW DurcanTM BrownCM McPhersonP LaflammeC 2023Antibody Characterization Report for Matrin-3 (2023)Zenodo10.5281/zenodo.10085229 ZhaCY BolívarSG AyoubiR McPhersonP LaflammeC NeuroSGC/YCharOS/EDDU collaborative group 2025Antibody Characterization Report for NEK1 (UniProt ID: Q96PY6) for use in western blotZenodo10.5281/zenodo.17955305 MoleónVR AyoubiR AlendeC FothouhiM RyanJ BolívarSG WorrallD DurcanTM BrownCM FrancisV FrancisV McPhersonPS LaflammeC 2025A guide to selecting high-performing antibodies for Optineurin (UniProt ID: Q96CV9) for use in western blot, immunoprecipitation, and immunofluorescenceF1000Research10.12688/f1000research.169966.1PMC1291417041716354 AyoubiR BolívarSG McPhersonPS LaflammeC 2025A guide to selecting high-performing antibodies for Senataxin (UniProt ID: Q7Z333) for use in western blotZenodo10.5281/zenodo.17253433 AlshafieW FotouhiM AyoubiR NicouleauM DurcanTM McPhersonPS LaflammeC 2021Antibody Characterization Report for Sigma non-opioid intracellular receptor 1Zenodo10.5281/zenodo.5644356 AyoubiR AlshafieW YouZ SouthernK McPhersonPS LaflammeC 2023Identification of high-performing antibodies for Superoxide dismutase [Cu-Zn] 1 (SOD1) for use in Western blot, immunoprecipitation, and immunofluorescenceF1000Research10.12688/f1000research.132952.2PMC1058262137860271 AyoubiR FotouhiM RyanJ ReintschW DurcanTM BrownC McPhersonPS LaflammeC 2022Antibody Characterization Report for SpatacsinZenodo10.5281/zenodo.7459431 AyoubiR AlshafieW ShlaiferI SouthernK McPhersonPS LaflammeC NeuroSGC/YCharOS/EDDU collaborative group 2024The identification of high-performing antibodies for Sequestosome-1 for use in Western blot, immunoprecipitation and immunofluorescenceF1000Research10.12688/f1000research.132628.2PMC1124008239006307 MoleónVR AlendeC FotouhiM AyoubiR LaflammeC NeuroSGC/YCharOS/EDDU collaborative group ABIF consortium 2025A guide to selecting high-performing antibodies for TAF15 (UniProt ID: Q92804) for use in western blot, immunoprecipitation, and immunofluorescenceF1000Research10.12688/f1000research.160371.1PMC1196609740182019 WorrallD AyoubiR FotouhiM SouthernK McPhersonPS LaflammeC NeuroSGC/YCharOS/EDDU collaborative group ABIF Consortium 2023The identification of high-performing antibodies for TDP-43 for use in Western Blot, immunoprecipitation and immunofluorescenceF1000Research10.12688/f1000research.131852.2PMC1028533437359785 AlshafieW FotouhiM ShlaiferI AyoubiR EdwardsAM DurcanTM McPhersonPS LaflammeC 2022Identification of highly specific antibodies for Serine/threonine-protein kinase TBK1 for use in immunoblot, immunoprecipitation and immunofluorescenceF1000Research10.12688/f1000research.124632.2PMC964714736415206 FotouhiM WorrallD AyoubiR SouthernK McPhersonPS LaflammeC NeuroSGC/YCharOS/EDDU collaborative group ABIF Consortium 2023A guide to selecting high-performing antibodies for RNA-binding protein TIA1 for use in Western Blot, immunoprecipitation and immunofluorescenceF1000Research10.12688/f1000research.133645.2PMC1102459638638178 FotouhiM AlshafieW ShlaiferI AyoubiR DurcanTM McPhersonPS LaflammeC 2022Antibody Characterization Report for Tubulin alpha-4A chainZenodo10.5281/zenodo.5903719 McDowellI AyoubiR FotouhiM SouthernK McPhersonPS LaflammeC NeuroSGC/YCharOS/EDDU collaborative group ABIF Consortium 2023The identification of high-preforming antibodies for Ubiquilin-2 for use in Western Blot, immunoprecipitation, and immunofluorescenceF1000Research10.12688/f1000research.131851.1PMC1028535337359784 BolívarSG AyoubiR AlendeC FothouhiM ShlaiferI McPhersonPS LaflammeC NeuroSGC/YCharOS/EDDU collaborative group ABIF Consortium 2024A guide to selecting high-performing antibodies for VAPB (UniProt ID: O95292) for use in western blot, immunoprecipitation, and immunofluorescenceF1000Research10.12688/f1000research.160226.1PMC1179976039916983 MoleónVR FotouhiM RyanJ WorrallD AyoubiR FrancisV McPhersonPS LaflammeC 2025A guide to selecting high-performing antibodies for VCP (UniProt ID: P55072) for use in western blot, immunoprecipitation, and immunofluorescenceF1000Research10.12688/f1000research.169502.1PMC1254737941142959 AyoubiR LaflammeC 2026ALS-RAP immunoblot dataset_chemiluminescent-based experimentZenodo10.5281/zenodo.18484385 AyoubiR LaflammeC 2026ALS-RAP immunoblot dataset_fluorescence-based experimentZenodo10.5281/zenodo.18484483

## Additional files

Supplementary file 1. List of 303 antibodies targeting ALS-RAP proteins evaluated in this study, together with the DOI linking to the corresponding antibody characterization reports.

Supplementary file 2. Sequences of synthetic ScFv antibodies.

MDAR checklist
