# Supplementary material for: A validated antibody toolbox for ALS research
Source: eLife. 2026 Nov 3;15:RP111349. doi: 10.7554/eLife.111349 (PMC13730523; doi:10.7554/eLife.111349)
Supplement: Peer review history

# Editor report: eLife Assessment

**DOI:** 10.7554/eLife.111349.3.sa0

**Authors:** Mychael V Lourenco

**Affiliations:** Universidade Federal do Rio de Janeiro Brazil

**Keywords:** Important, Solid

Overall, this is an **important** article that delivers an invaluable community resource. The execution is relatively simple, but the evidence of this resource is **solid**. The effort invested in generating the knockout lines that for validation experiments is a clear strength of the study, but it does not substitute for a careful evaluation of the same reagents in the end-users’ specific protocols before making any biological statement.

# Referee report: Reviewer #1 (Public review)

**DOI:** 10.7554/eLife.111349.3.sa1

Summary:

The authors address the lack of validated tools for detection and quantification of proteins associated with amyotrophic lateral sclerosis (ALS) through an extensive screening of 303 commercially available antibodies to 33 protein targets. Their ALS-Reproducible Antibody Platform (ALS-RAP) delivers a validated antibody toolbox for ALS research, which will provide an advantageous starting point for researchers in this field. Ayoubi R. et al. showcase the characterization workflow, presenting as an example the characterization of antibodies targeting Galectin-1, encoded by the LGALS1 gene. A selection of these antibodies was also used to profile protein levels across human induced pluripotent stem cell (iPSC)-derived and primary neurological cell types, and the fundings support that ALS disease mechanism involves both neuronal and glial cells.

Strengths:

The knockout (KO)-based approach is definitively the major strength of this study, providing a high level of confidence in the data collected in human induced pluripotent stem cell (iPSC)-derived and primary neurological cell types. Important is also the focus on renewable reagents (monoclonal and recombinant antibodies). The extensive characterization of this set of antibodies will benefit any scientist interested in any of the 33 targets proteins, even in fields other than neuroscience.

The authors perform an interesting protein profiling study assessing 27 proteins, comparing RNA and protein expression data, and using two independent WB preparations of the same cell types. The conclusions that can be drawn from this first assessment might not be final, but data are compelling, because they have been collected with reliable and validated antibodies.

Another strength of this work is the data dissemination strategy, which includes the Only Good Antibodies (OGA) platform, where YCharOS data are curated and presented in an easy and intuitive manner that facilitates antibody selection by the end user for WB, IP and IF applications.

The authors mentioned the development of single-chain variable fragment (scFv) recombinant antibodies raised by the SGC against the six proteins (ANXA11, OPTN, MATR3, PFN1, UBQLN2 and VCP) that had limited renewable antibodies that are commercially available. The development was optimized to generate antibodies particularly suitable for IP, and the clone selection process was carried out using IP coupled to mass spectrometry. They provide details on the screening process for the SGC-generated scFv antibodies, plus share that scFv sequences.

Weakness:

The protein profiling study is limited to WB data, and the authors did not provide any explanation on why there was no integration with IP and IF data, not even for those targets that have validated antibodies. Also, not all the cell types have been screened by chemiluminescence-based detection and by fluorescence-based WB, and the authors do not elaborate on the reason for such choice. Technical and practical considerations for such decisions could have been briefly described.

# Referee report: Reviewer #2 (Public review)

**DOI:** 10.7554/eLife.111349.3.sa2

Overall, this is a solid manuscript that delivers an important community resource. The execution is relatively simple, but the value is real, the work is rigorously performed, and the open dissemination through Zenodo, the F1000Research YCharOS Gateway and OGA is well executed. The effort invested in generating the knockout lines for validation experiments is a clear strength of the study.

Comments on revised version.

I thank the authors for their responses. A number of the smaller points I raised are now resolved. However, I am disappointed to see that the more substantial concerns, which relate to the cell-type expression claims running through the Results and the Discussion, remain unaddressed. My original point was that these claims are not quantitatively supported and that the identity of the iPSC-derived cell types used to make them has not been documented, and I do not think this revision changes that. Most of what I asked for was quantification of experiments the authors have already performed, or quality control on the cell types themselves, and this has been answered with scope arguments rather than with data. The other points I originally raised remain.

I give further details on my two main remaining concerns below:

(1) The cell-type expression claims across the manuscript are still supported by qualitative Western blot images only. These include statements such as "higher signal in glial populations," "enriched in neuronal lineages," "predominantly detected in microglial populations," and the "convergence on multicellular disease mechanisms" narrative in the Discussion. I understand the authors' decision to keep ALS-RAP as a tool and resource paper rather than expand the experimental work. However, if that is the framing, the biological interpretations of these expression data need to be removed. In their response, the authors themselves acknowledge that "quantification across multiple biological replicates would be required to establish definitive differences in protein abundance between neuronal and glial cell types." As long as this quantification is not provided, every claim of cell-type-specific expression should be removed from the Abstract, Results and Discussion, and the data presented as a descriptive survey.

(2) Related to the previous point, I do not think that quality control of the non-microglia iPSC lineages is beyond the scope of the study, as argued in the response. For ALS-RAP to be adopted as a resource, other laboratories need to trust that the authors' iPSC-derived neurons, astrocytes and oligodendrocytes are really neurons, astrocytes and oligodendrocytes. The fact that the differentiation protocols have been previously published does not confirm that they have been correctly implemented here, and this is precisely the kind of quality control that should be documented in the paper. In their response, the authors list the single markers already shown in Figure S3 (ISL1, TH, MBP, GFAP, CD44) as the answer to my concern, but a single positive marker per lineage does not establish purity.

# Author comment: Author response

**DOI:** 10.7554/eLife.111349.3.sa3

**Authors:** Riham Ayoubi, Emma J MacDougall, Ian McDowell, Michael S Biddle, Bárbara T Ferreira, CongYao Zha, Marie-France Dorion, Jay Ross, Sara Gonzalez Bolivar, Vera Ruiz Moleon, Charles Alende, Vincent Francis, Maryam Fotouhi, Mathilde Chaineau, Carol X Chen, Valerio E Piscopo, Vincent Soubannier, Irina Shlaifer, Zhipeng You, Michael Nicoleau, Tracy Keates, Wen Hwa Lee, Brian D Marsden, Leonidas Koukouflis, Edvard Wigren, Carolyn A Marks, Luke M Healy, Patrick A Dion, Guy A Rouleau, Edward A Fon, Harvinder Virk, Susanne Gräslund, Opher Gileadi, Aled M Edwards, Thomas M Durcan, Peter S McPherson, Carl Laflamme

**Affiliations:** McGill University Montreal Canada; McGill University Montreal Canada; McGill University Montreal Canada; University of Leicester Leicester United Kingdom; University of Leicester Leicester United Kingdom; McGill University Montreal Canada; McGill University Montreal Canada; Stanford Medicine Standord United States; McGill University Montreal Canada; McGill University Montreal Canada; McGill University Montreal Canada; McGill University Montreal Canada; Montreal Neurological Institute, McGill University Montreal Canada; McGill University Montreal Canada; Montreal Neurological Institute and Hospital, McGill University Montreal Canada; Montreal Neurological Institute and Hospital Montreal Canada; Montreal Neurological Institute and Hospital Montreal Canada; Montreal Neurological Institute, McGill University Montreal Canada; McGill University Montreal Canada; McGill University Montreal Canada; University of Oxford Oxford United Kingdom; University of Oxford Oxford United Kingdom; University of Oxford Oxford United Kingdom; University of Oxford Oxford United Kingdom; Karolinska Institutet Sockholm Sweden; Karolinska Institutet Sockholm Sweden; McGill University Montreal Canada; McGill University Montreal Canada; McGill University Montreal Canada; Montreal Neurological Institute and Hospital, McGill University Montreal Canada; University of Leicester Leicester United Kingdom; Karolinska Institutet Sockholm Sweden; Karolinska Institutet Stockholm Sweden; University of Toronto Toronto Canada; McGill University Montreal Canada; Montreal Neurological Institute, McGill University Montreal Canada; McGill University Montreal Canada

The following is the authors’ response to the original reviews.

**Public Reviews:**

**Reviewer #1 (Public review):**

(1) The authors mentioned the development of single-chain variable fragment (scFv) recombinant antibodies raised by the SGC against the six proteins (ANXA11, OPTN, MATR3, PFN1, UBQLN2 and VCP) that had limited renewable antibodies that are commercially available. The development was optimized to generate antibodies particularly suitable for IP, and the clone selection process was carried out using IP coupled to mass spectrometry. Even though the generation of these novel reagents is not the focus of this work, the authors do not provide any data on this aspect.

The scFv reagents represent an important component of this resource and warranted additional description. In the revised manuscript, we expanded the section describing their origin, selection strategy, and intended use. We also added an updated reference describing the scFv discovery methodology, together with a brief summary of the workflow. In addition, the sequences of all six scFvs (ANXA11, OPTN, MATR3, PFN1, UBQLN2, and VCP) are now provided in Supplementary Table 2. During the revision process, we also identified that two scFv plasmids were missing from Add gene; these have now been deposited, making all six reagents and their sequences publicly available.

(2) The protein profiling study is limited to WB data, and the authors did not provide any explanation on why there was no integration with IP and IF data, not even for those targets that have validated antibodies. Also, not all the cell types have been screened by chemiluminescence-based detection and by fluorescence-based WB, and the authors do not elaborate on the reason for such a choice.

ALS-RAP was conceived as an open science resource for the ALS community. Given the breadth of the project (33 ALS-associated proteins), it is unrealistic for a single laboratory to investigate the biochemical and cellular biology of every target. We therefore generated and openly disseminated antibody characterization data across three complementary applications—western blot (WB), immunoprecipitation (IP), and immunofluorescence (IF)—to provide the community with validated tools that can support diverse downstream studies, including protein abundance, protein interactions, and subcellular localization.

The primary objective of the protein profiling study presented in this manuscript, however, was to compare relative protein abundance across multiple neuronal and glial cell types. Western blotting was selected because it provides a robust and semi-quantitative measurement of protein abundance across biological samples, making it well suited for systematic comparisons between cell types. In contrast, IP and IF were primarily included as antibody validation assays and as community resources for future mechanistic studies rather than for quantitative expression profiling.

Generating the large quantities of iPSC-derived neuronal and glial cells required to profile 33 ALS-associated proteins is technically demanding and resource-intensive. Consequently, a limited number of protein-cell type combinations are absent from one of the two detection modalities because insufficient lysate remained for repeat analyses. Importantly, every protein was evaluated across the complete panel of neurological cell types in at least one western blot detection modality. In most cases, the omitted samples corresponded to cell types in which the target protein was undetectable or expressed at very low levels in the initial experiment.

**Reviewer #2 (Public review):**

(1) The rationale for the selection of these 33 genes is insufficient. The authors lean on the Nijs & VanDamme classification and on PubMed entry counts, but the number of PubMed entries is not a meaningful criterion for what constitutes an important ALS protein - some of the most disease-relevant genes are precisely those with fewer publications, while heavily cited genes such as CAV1 carry weak ALS-specific evidence. The authors should provide a more transparent and biologically motivated rationale for inclusion and exclusion (ClinGen evidence tier, replicated GWAS signals, large meta-analyses, ALSoD) and explain why specific risk genes outside this list were not part of ALS-RAP.

We thank the reviewer for this comment and apologize if our rationale for target selection was not sufficiently clear. We agree that the number of PubMed entries should not be interpreted as a criterion for inclusion. Rather, we included this metric as a descriptive measure of how extensively each gene has been studied in the context of ALS. Specifically, comparing the number of publications for Gene versus Gene + ALS provides an indication of the relative attention a target has received within the ALS field, but it was not used to prioritize genes for inclusion.

The selection of the 33 ALS-RAP targets was instead based on established genetic evidence for disease association. Specifically, we prioritized ALS-associated genes supported by replicated human genetic studies, including rare variants with high, intermediate, or modest effect sizes, following the classification framework proposed by Nijs and Van Damme. As described in the manuscript, 25 of the 33 targets have also been independently curated by the Clinical Genome Resource (ClinGen) ALS Gene Curation Expert Panel and classified as having evidence supporting an association with ALS (Table 1). The remaining genes were selected based on published human genetic studies and emerging evidence supporting their relevance to ALS biology.

(2) "107 of 231 (46%) demonstrated specific target staining in IF." The criteria used to define "specific target staining" at the IF level are not stated. From the Galectin-1 example, the mosaic WT/KO strategy provides a binary readout, but for proteins with low expression, weak punctate staining or unusual subcellular distributions, a single threshold is unlikely to capture specificity uniformly across 231 antibodies.

We thank the reviewer for highlighting this omission. We agree that the criteria used to classify antibodies in each application should be explicitly stated. In the revised manuscript, we now define the classification criteria for Western blot (WB), immunoprecipitation (IP), and immunofluorescence (IF) in the Methods section. Briefly, WB antibodies were considered target-selective when the predominant immunoreactive band at the expected molecular weight was substantially reduced or absent in the corresponding knockout (KO) lysate. IP antibodies were considered successful when they specifically enriched the target protein from wild-type lysates, as assessed by immunoblotting of the immunoprecipitated material. For IF, antibodies were considered specific when the mean fluorescence intensity in wild-type cells was at least 1.5-fold higher than that measured in KO cells using the mosaic WT/KO assay. A minimum of 250 WT and 250 KO cells were quantified for each antibody. These criteria were applied uniformly across all antibodies to provide a standardized and objective benchmarking framework. We have also added references to the detailed YCharOS characterization workflows previously described in Ayoubi et al., Nature Protocols (2025).

(3) Several claims in the manuscript depend on differential protein abundance across cell types. As presented, these claims are supported by qualitative Western blot images only. They should be substantiated by quantification across multiple biological replicates.

The expression patterns observed were reproducible across two independent batches of iPSC-derived neuronal and glial cell types generated using the same differentiation protocols. In addition, several proteins enriched in iPSC-derived microglia also showed enrichment in primary human microglia and monocyte-derived macrophages, providing independent support for these observations.

We agree that quantification across multiple biological replicates would be required to establish definitive differences in protein abundance between neuronal and glial cell types. However, this was not the objective of the present study. Rather, our goal was to perform an initial survey of ALS-associated protein expression using rigorously validated antibodies and to identify expression patterns that may warrant further investigation.

ALS-RAP was conceived as an open science resource for the ALS community. Given the breadth of the project (33 ALS-associated proteins), we do not envision that a single laboratory can comprehensively define the expression, localization, and function of every target across all relevant cell types, tissues, and disease states. Instead, we provide validated reagents together with an initial characterization of protein expression in well-defined iPSC-derived neuronal and glial cell models to enable and accelerate future studies by the broader research community.

(4) This manuscript represents a unique opportunity to address antibody recognition of splicing variants, which is something of considerable value to the community. For each target, the predicted isoforms in Ensembl could be cross-referenced against the observed bands, and the pattern of bands compared across cell types could be informative about which isoforms each antibody captures. This would convert ambiguous "extra bands" into useful biological information and would substantially increase the value of the resource. I strongly encourage the authors to include this analysis.

We agree that defining which protein isoforms are recognized by individual antibodies would be valuable to the research community. However, we believe that such an analysis would be difficult to interpret and potentially misleading based on the data generated in the present study.

All antibodies were initially characterized in a common cancer cell line, and the best-performing reagents were subsequently used to profile ALS-associated proteins in iPSC-derived neuronal and glial cell types. Additional bands observed in neurological cells, but not in the original characterization cell line, may reflect several nonmutually exclusive possibilities, including cross-reactivity with proteins specifically expressed in neurological cells, recognition of cell type-specific splice isoforms, or post-translationally modified forms of the target protein.

Without knockout validation in each neurological cell type, these possibilities cannot be distinguished confidently.

In addition, protein migration on SDS-PAGE frequently differs from the theoretical molecular weight predicted from amino acid sequence alone. Consequently, assigning individual immunoreactive bands to Ensembl-predicted isoforms based solely on apparent molecular weight would be speculative and could lead to incorrect conclusions. We therefore believe that a rigorous isoform analysis would require complementary experimental approaches beyond the scope of the present resource study.

(5) The iPSC-derived microglia receive a comprehensive QC panel (IBA1/PU.1 IF, CD45/CD11b flow, qRT-PCR for nine canonical markers; Figure S4), which allows the reader to assess culture purity. The other iPSC-derived lineages - motor neurons, dopaminergic neurons, oligodendrocytes and astrocytes- are validated by a single marker each in WB (Figure S3) without purity quantification. Given that several conclusions of the manuscript rest on the cell-type-specific detection of ALS-associated proteins, equivalent quality control should be performed for the other lineages so that the reader can evaluate the purity of each preparation.

We thank the reviewer for this important comment and agree that documenting the identity of iPSC-derived cell populations is essential given their inherent heterogeneity. The differentiation protocols used in this study follow well-established, published methods that have been extensively validated by their developers and the broader stem cell community. We confirmed the identity of each differentiated lineage using established lineage-specific markers, including ISL1 for motor neurons, TH for dopaminergic neurons, SATB2 and CTIP2 for cortical neurons, MBP for oligodendrocytes, GFAP and CD44 for astrocytes, and CD11b and Iba1 for microglia.

We agree that comprehensive quantitative purity analyses for every differentiated lineage would further strengthen the study. However, we believe that these experiments fall beyond the scope of the present work, whose primary objective was to establish a validated antibody resource rather than to optimize or benchmark differentiation protocols. We also note that our conclusions are intentionally limited to broad protein expression patterns rather than definitive quantitative comparisons between cell types.

Finally, the inclusion of primary human microglia, fetal astrocytes, and monocyte-derived macrophages provides an independent layer of biological validation. The concordance of expression patterns observed between these primary cells and the corresponding iPSC-derived populations supports the robustness of our principal observations.

(6) The robustness of the resource would be substantially increased by validating at least a subset of the targets in a second iPSC background, in at least some of the cell types analysed.

We agree that validating antibody performance and protein expression across multiple iPSC genetic backgrounds would further strengthen the resource. However, we believe that this objective falls beyond the scope of the present study, whose primary aim was to establish a rigorously validated antibody toolbox for ALS-associated proteins and to provide an initial survey of their expression in representative neuronal and glial cell types.

We envision ALS-RAP as the foundation of a broader community effort. Rather than comprehensively evaluating all 33 proteins across multiple genetic backgrounds and tissues, our goal is to provide validated, renewable reagents that will enable such studies by the broader ALS research community.

(7) The newly developed SGC scFv antibodies are arguably the most novel reagent contribution of this manuscript, yet they receive a single sentence in the body of the paper. A more thorough description is warranted.

We agree that the scFv reagents represent an important component of this resource and warranted additional description. In the revised manuscript, we expanded the section describing their origin, selection strategy, and intended use. We also added an updated reference describing the scFv discovery methodology, together with a brief summary of the workflow. In addition, the sequences of all six scFvs (ANXA11, OPTN, MATR3, PFN1, UBQLN2, and VCP) are now provided in Supplementary Table 2. During the revision process, we also identified that two scFv plasmids were missing from Addgene; these have now been deposited, making all six reagents and their sequences publicly available.

(8) Accessibility of the resource through Zenodo is not straightforward - the reader currently has to navigate to individual antibody characterization reports one by one to extract recommendations for a given target. While the use of an established public repository is important for permanence, a dedicated ALS-RAP website with an interactive, searchable interface - filterable by target, application, host species and clonality - would meaningfully improve uptake. The relationship between such a portal and the existing OGA platform should also be clarified.

We agree that navigating individual antibody characterization reports on Zenodo can be cumbersome for end users. This challenge was one of the motivations behind the development of the Only Good Antibodies (OGA) platform, which curates YCharOS antibody characterization data, including the ALS-RAP dataset, into an interactive and searchable interface. OGA allows users to identify antibodies by target, application, host species, clonality, and other relevant attributes, while providing direct links to the complete characterization reports archived on Zenodo. In this way, Zenodo serves as the permanent open repository for the complete datasets, whereas OGA provides a user-friendly interface for data exploration and antibody selection.
